# Multimodality 3D image fusion with live fluoroscopy reduces radiation dose during catheterization of congenital heart defects

**DOI:** 10.3389/fcvm.2023.1292039

**Published:** 2024-01-11

**Authors:** Dimitri Buytaert, Kristof Vandekerckhove, Joseph Panzer, Laurence Campens, Klaus Bacher, Daniël De Wolf

**Affiliations:** ^1^ Department of Human Structure and Repair, Ghent University, Ghent, Belgium; ^2^ Department of Paediatric Cardiology, Ghent University Hospital, Ghent, Belgium; ^3^ Department of Cardiology, Ghent University Hospital, Ghent, Belgium; ^4^ Department of Paediatric Cardiology, Brussels University Hospital, Jette, Belgium

**Keywords:** congenital heart disease, cardiac catheterization, multimodality image fusion, radiation exposure, contrast media, 3D guidance, radiation dose structured report

## Abstract

**Introduction:**

Imaging fusion technology is promising as it is radiation and contrast sparing. Herein, we compare conventional biplane angiography to multimodality image fusion with live fluoroscopy using two-dimensional (2D)–three-dimensional (3D) registration (MMIF_2D−3D_) and assess MMIF_2D−3D_ impact on radiation exposure and contrast volume during cardiac catheterization of patients with congenital heart disease (CHD).

**Methods:**

We matched institutional MMIF_2D−3D_ procedures and controls according to patient characteristics (body mass index, age, and gender) and the seven procedure-type subgroups. Then, we matched the number of tests and controls per subgroup using chronological ordering or propensity score matching. Subsequently, we combined the matched subgroups into larger subgroups of similar procedure type, keeping subgroups with at least 10 test and 10 control cases. Air kerma (AK) and dose area product (DAP) were normalized by body weight (BW), product of body weight and fluoroscopy time (BW × FT), or product of body weight and number of frames (BW × FR), and stratified by acquisition plane and irradiation event type (fluoroscopy or acquisition). Three senior interventionists evaluated the relevance of MMIF_2D−3D_ (5-point Likert scale).

**Results:**

The Overall group consisted of 54 MMIF_2D−3D_ cases. The combined and matched subgroups were pulmonary artery stenting (Stent_PUL_), aorta angioplasty (Plasty_AO_), pulmonary artery angioplasty (Plasty_PUL_), or a combination of the latter two (Plasty). The FT of the lateral plane reduced significantly by 69.6% for the Overall MMIF_2D−3D_ population. AK_BW_ and DAP_BW_ decreased, respectively, by 43.9% and 39.3% (Overall group), 49.3% and 54.9% (Plasty_AO_), and 36.7% and 44.4% for the Plasty subgroup. All the aforementioned reductions were statistically significant except for DAP_BW_ in the Overall and Plasty (sub)groups. The decrease of AK_BW_ and DAP_BW_ in the Stent_PUL_ and Plasty_PUL_ subgroups was not statistically significant. The decrease in the median values of the weight-normalized contrast volume (CMC_BW_) in all five subgroups was not significant. Cardiologists considered MMIF_2D−3D_ very useful with a median score of 4.

**Conclusion:**

In our institution, MMIF_2D−3D_ overall enabled significant AK_BW_ reduction during the catheterization of CHD patients and was mainly driven by reduced FT in the lateral plane. We observed significant AK_BW_ reduction in the Plasty and Plasty_AO_ subgroups and DAP_BW_ reduction in the Plasty_AO_ subgroup. However, the decrease in CMC_BW_ was not significant.

## Introduction

Congenital heart disease (CHD) is the most common birth defect (1–3). Cardiac catheterization has improved management of CHD patients, and owing to the increased reliability and advancements in technology, the number, types, and complexity of cardiac catheterizations have increased dramatically in recent years (4–7). In addition, multiple staged or repeated procedures are required during the lifetime of patients with complex CHD lesions, which significantly increases their cumulative radiation exposure (1, 8, 9). For a given radiation dose, children are three to four times more susceptible to radiation-induced malignancies. Moreover, their smaller body size results in less attenuation, and therefore relatively higher doses for deeper organs, and a relatively larger fraction of their body is exposed to the direct x-ray beam (10–14). Furthermore, operators are often near the patient, thereby increased patient exposure results in increased occupational exposure. Consequently, optimizing radiation exposure of cardiac catheterization in CHD is needed. Image fusion of a three-dimensional (3D) reconstruction of volumetric imaging datasets with live fluoroscopy has the potential to reduce iodinated contrast volume and radiation dose (15–17). In the last decade, 3D rotational angiography image fusion (3DRA-IF) has been more frequently used in CHD, where a 3D roadmap is obtained from a tomographic acquisition, which is subsequently overlaid on the live fluoroscopy images acquired by the same x-ray modality (16, 18–22).

More recently, multimodality image fusion (MMIF) has become available. With the latter technology, a 3D roadmap can be obtained from preprocedural imaging like computed tomography (CT) or magnetic resonance imaging (MRI) (15, 23–26). After segmentation, the 3D roadmap is registered with live fluoroscopy using either two fluoroscopy or cinegraphy acquisitions in different projections, i.e., 2D–3D registration (MMIF_2D−3D_), or using a 3DRA acquisition, i.e., 3D–3D registration (MMIF_3D−3D_) (26). With MMIF_2D−3D_ only two fluoroscopy exposures are sufficient to perform registration with the volumetric dataset, resulting in nearly radiation-free registration (24, 26, 27).

The goal of the current study is to assess the impact of MMIF_2D−3D_ on radiation exposure and contrast volume levels during cardiac catheterization in CHD patients.

## Methods

The procedural and patient data included in this study were acquired from the institutional database on congenital cardiac catheterizations performed between January 2015 and October 2019. Structural interventions such as closure of atrial septal defects (ASD), ventricular septal defects (VSD), patent foramen ovale (PFO) and patent ductus arteriosus (PDA) were excluded since they were ultrasound guided, without the use of ultrasound-fluoroscopy image fusion. All procedures were performed with a biplane AlluraClarity FD20/FD10 system (Philips Healthcare, Best, The Netherlands). The diagonal of the frontal imaging detector was double the size of the lateral imaging detector. The imaging protocols available on the biplane x-ray system are split by weight in two groups, i.e., ≤40 and >40 kg. In both weight groups, the corresponding lowest dose imaging protocols are selected by default. The operators are used to working with the lowest dose settings and switch to higher dose settings only if they deem it necessary.

### Multimodality image fusion

**Figure 1:**
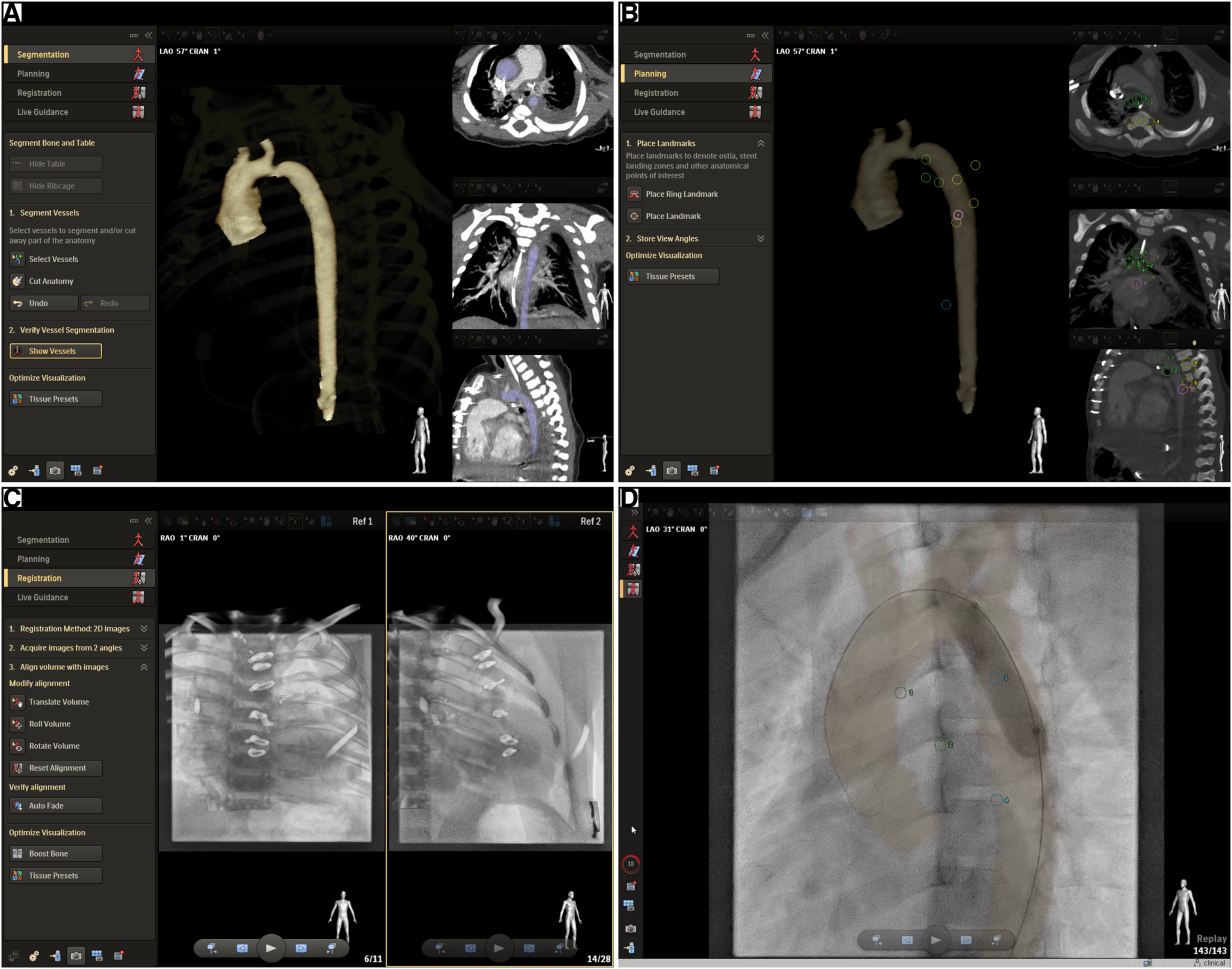
Multimodality image fusion from CT or MRI using VesselNavigator. The screenshots showcase an aortic coarctation MMIF_2D−3D_ case and the four-step workflow followed to achieve registration of the 3D volumetric data with the live fluoroscopy imaging. (**A**) Segmentation: The target anatomy is segmented from the volumetric dataset. In this case a preprocedural CT exam was available. (**B**) Planning: The case can be prepared by placing anatomical landmarks and measurements. Optimal viewing angles can be saved before the procedures and can be recalled and synchronized with the C-arm during the procedure. The screenshot shows anatomical landmarks that were added for further fine-tuning of the registration during Live Guidance, i.e., the tracheal carina, the inferior edge of the left and right main bronchus, alternately the left and right edge of four consecutive thoracic vertebrae starting from the level of the tracheal carina (T4/T5), the apex, and one landmark in a sagittal view on the anterior edge of one of the vertebrae between T4 and T8. (**C**) Registration: The volumetric dataset, in this case a CT volume, should be transformed to the coordinate system of the x-ray fluoroscopy system. Structures that are easily visible on both CT and fluoroscopy, in this case the vertebrae and ribs from CT, are volume rendered transparently on top of two fluoroscopy projections at least 45° apart. The CT volume can then be manually aligned with the fluoroscopy image in both projections. In the current study the two projections were always a frontal and lateral low-dose fluoroscopy exposure. (**D**) Live Guidance: the CT volume is now synchronized with the x-ray angiography system and it will follow any table and C-arm movement accordingly. The segmented anatomy is now transparently overlaid on top of the x-ray fluoroscopy or acquisition in real-time. This screenshot shows a balloon dilatation of an aortic coarctation.

During the study period, we applied MMIF_2D−3D_ when CT-scan or MRI imaging of sufficient quality was available for 3D reconstruction and used the conventional biplane 2D angiography (2DA) in the absence of preprocedural imaging. The MMIF_2D−3D_ approach consisted of a four-step process and was performed using the VesselNavigator software (Philips Healthcare, Best, The Netherlands) that has been described previously in the literature (28). Briefly, the anatomy of interest was segmented. Then, the anatomical landmarks visible on both live fluoroscopy and volumetric datasets (CT or MRI) were indicated on the volumetric dataset. Afterward, two short 2D fluoroscopy projections were performed to match the position of the preprocedural 3D volume with the periprocedural anatomical position. The final step is live guidance of the procedure with the registered 3D volume transparently overlaid on top of the live fluoroscopy, during which the registration can be optimized further. The process is visualized in Figure 1, showcasing one aortic coarctation MMIF_2D−3D_ case.

### Matching

**Figure 2:**
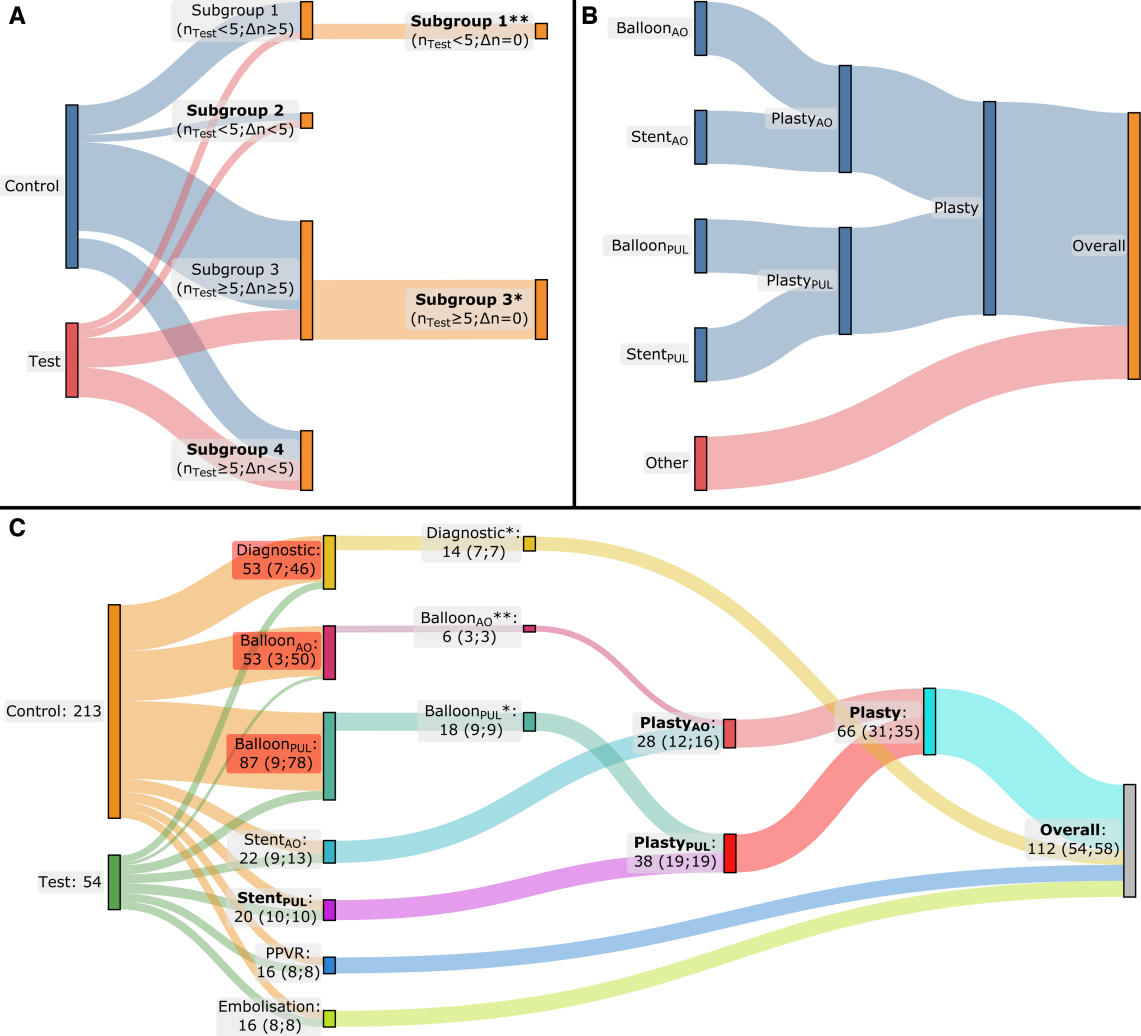
Flow diagrams of multiphase matching. The applied method is depicted in (**A**) and (**B**), while the result for our patient cohort is depicted in (**C**). (**A**) During the first phase of the matching process, the cases are grouped by procedure type, resulting in four potential situations: a procedure-type subgroup where n_Test_ < 5 and Δn ≥ 5 (Subgroup 1), a second subgroup where n_Test_ < 5 and Δn < 5 (Subgroup 2), a third subgroup where n_Test_ ≥ 5 and Δn ≥ 5 (Subgroup 3), and a fourth and final one where n_Test_ ≥ 5 and Δn < 5 (Subgroup 4). When Δn ≥ 5, a second matching phase was performed to end up with the same number of cases in the test and control subgroups (Δn = 0): propensity score matching was performed based on BMI, age, gender, and number of treated lesions (the latter only in case of angioplasty) when n_Test_ ≥ 5 (Subgroup 3*). Alternatively, when n_Test_ < 5, the second matching phase was conducted by ordering the control subgroup chronologically and retaining only the last n_Test_ cases from the control subgroup (Subgroup 1**). (**B**) The subgroups of balloon dilatation and stenting in the aorta and pulmonary artery were combined into angioplasty subgroups for the aorta and pulmonary artery, respectively, and subsequently into one single angioplasty subgroup. Finally, all the subgroups were combined into one single group. (**C**) The result of the matching process: after the initial matching phase, a second matching phase was necessary for the Diagnostic and Balloon_PUL_ subgroups by applying propensity score matching for the Diagnostic and Balloon_PUL_ subgroups (*) and chronological ordering for the Balloon_AO_ subgroup (**). Finally, statistical analysis was performed only for the subgroups with ≥10 cases in both the test and control groups, these subgroups are formatted in bold text; n_Test_, number of test cases in the subgroup; Δn, absolute difference between the number of test cases and the control cases in the corresponding subgroup.

To reduce bias, we applied a multiple step matching process based on procedure type and patient characteristics [body mass index (BMI), age, and gender]. Initially, we subdivided the MMIF_2D−3D_ procedures into seven subgroups: diagnostic, balloon dilatations in the aorta (Balloon_AO_), balloon dilatations in the pulmonary artery (Balloon_PUL_), stenting in the aorta (Stent_AO_), stenting in the pulmonary artery (Stent_PUL_), percutaneous pulmonary valve replacements (PPVR), and embolization. Subsequently, for each test group we selected a corresponding control subgroup from conventional CHD procedures of the same type performed during that time period. If the difference in the number of cases between the test and control subgroups (Δn) was less than 5, then we considered these subgroups as matched. When Δn ≥ 5 and the number of cases in the test subgroup (n_Test_) was larger than 5, we conducted a propensity score matching of 1:1 on BMI, age, gender, and number of treated lesions (considered only for angioplasty procedures). In addition, if n_Test _< 5, we selected the latest n_Test_ cases from the chronologically ordered control subgroup as the matched control subgroup. After subgroup matching, we combined the subgroups of balloon dilatation and stenting into angioplasty subgroups for each respective region (Plasty_AO_ and Plasty_PUL_) before finally combining all subgroups into one Overall group. Eventually, only the subgroups with a minimum of 10 procedures each in both the test and control cohorts were included for statistical analysis. The matching process is illustrated in Figure 2.

### Radiation exposure data

We collected radiation exposure parameters from the Digital Imaging and Communications in Medicine (DICOM) Radiation Dose Structured Reports (RDSR) generated by the system at the end of each procedure. The parameters included cumulative dose area product (DAP), cumulative air kerma (AK), fluoroscopy time (FT), number of cinegraphy acquisitions, and number of cinegraphy frames (FR). Subsequently, DAP and AK were grouped by irradiation event type [fluoroscopy (F) and cinegraphy acquisition (A)], by acquisition plane [frontal (A) and lateral (B)], or by both. FT and FR were also grouped by acquisition plane.

**Figure 3:**
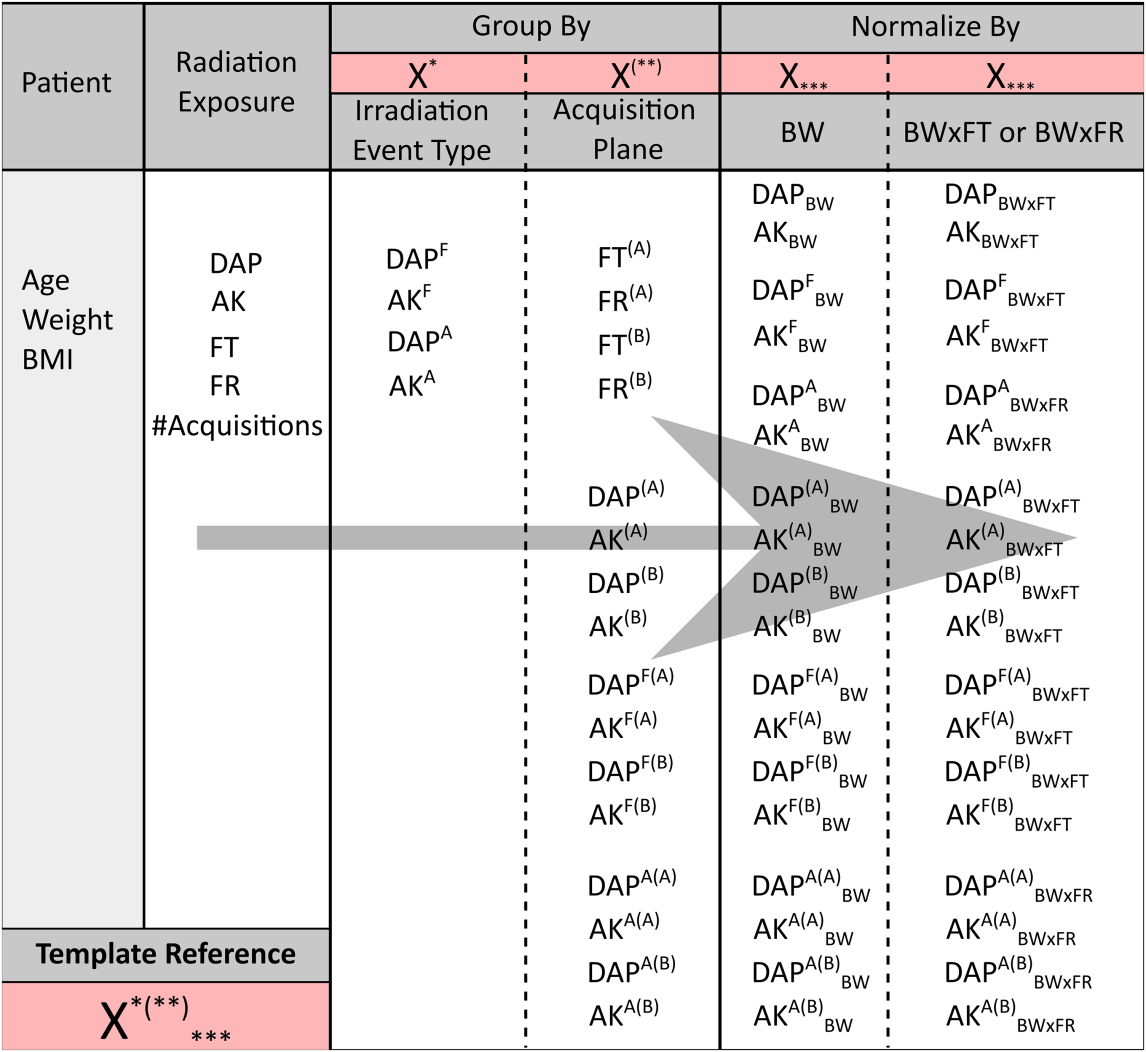
The different patient and radiation exposure parameters collected for the current study. From left to right, starting from the radiation exposure parameters, the latter parameters were grouped by either irradiation event type (fluoroscopy F or cinegraphy acquisition A) or by acquisition plane [frontal (A) or lateral (B) plane] or by both. The overall parameters and grouped parameters were subsequently normalized by either BW or BW × FT, except for DAP^A^ and AK^A^. The latter ones and their corresponding parameters grouped by plane were normalized by BW × FR instead of BW × FT. A template notation is given in the bottom left of the figure, e.g., X^*(**)^_***_. X refers to DAP, AK, FT, or FR. The first superscript *, i.e., either F or A, indicates grouping by fluoroscopy or acquisition, respectively. A missing first superscript * refers to the overall (F + A) DAP and AK. The second superscript between brackets (**), i.e., either (A) or (B), indicates grouping by the frontal or lateral plane, respectively. A missing second superscript (**) refers to the overall [(A) + (B)] DAP and AK of both acquisition planes combined. A missing subscript *** refers to the overall DAP and AK without normalization.

As proposed by the European Guidelines on Diagnostic Reference Levels (DRLs) for Paediatric Imaging, we looked at DAP and AK normalized by body weight (DAP_BW_ and AK_BW_) and by the product of body weight and fluoroscopy time (DAP_BW × FT_ and AK_BW × FT_) (6). For radiation exposure associated only with cinegraphy acquisition, the product of body weight and the number of frames (BW × FR) was used for normalization (DAP^A^_BW × FR_ and AK^A^_BW × FR_). All the collected and calculated radiation exposure parameters are summarized in Figure 3.

### Questionnaire

The three senior experienced pediatric interventional cardiologists performing all interventions included in this study were questioned for subjective evaluations of the MMIF_2D−3D_ cases. All catheterizations were reviewed on the Picture Archiving and Communication System (PACS) by the interventionists before filling out the three-question assessment. The first question was: “*Assuming you would have performed this case using conventional biplane 2DA, are there any images missing? If yes, please state the number of missing acquisitions, their required projection angles, and if these would have been single- or bi-plane acquisitions*”. We reported the number of missing angiographies as the number of spared acquisitions by the MMIF_2D−3D_ guidance. Interventionists follow an institutional standard protocol concerning contrast media consumption (CMC) per BW administered for each acquisition. Therefore, they were asked to estimate the spared CMC_BW_ associated with the spared acquisitions. Subsequently, screenshots of the 3D overlay registered with the fluoroscopy were shown next to the angiography images on the PACS and the second question was: “*How many angiographies would you consider redundant due to the availability of the MMIF_2D−3D_ guidance?*” They were also asked to estimate the CMC associated with each redundant acquisition. The third question was: “*Grade the relevance of MMIF_2D−3D_ guidance using the following Likert scale: 1 = Misleading, 2 = Not Useful, 3 = Useful, 4 = Very Useful, 5 = Essential*”.

### Statistical analysis

For normally distributed variables, significance testing of differences between MMIF_2D−3D_ and conventional 2DA was performed using an independent samples *t*-test with a 95% two-sided CI. A Mann–Whitney *U* test was performed in case of non-normality. The variables were summarized using median, 25th percentile (Q1), and 75th percentile (Q3). Comparison of categorical parameters was done using a χ^2^ test for association, also applied with a 95% CI. Interobserver agreement is assessed using Cohen's kappa. All statistical analyses were performed using IBM SPSS Statistics 28 (IBM Corp., Armonk, NY, USA).

## Results

Between January 2015 and October 2019, a total of 420 catheterizations were conducted. Among these procedures, structural interventions for conditions such as ASD, VSD, PFO, and PDA accounted for 153 cases. Of the 267 remaining procedures, 54 and 213 were linked to the test (MMIF_2D−3D_-guided catheterizations) and control groups, respectively. The following results only concern significant differences, unless explicitly mentioned otherwise.

### Matching

Figure 2C presents the outcome of the matching process. After initial matching, a subsequent matching phase was conducted for the Diagnostic, Balloon_AO_ and Balloon_PUL_ subgroups. The Diagnostic and Balloon_PUL_ subgroups underwent propensity score matching, while chronological ordering was used for Balloon_AO_. Five procedure type (sub)groups were selected for analysis after completing the multiphase matching process: Stent_PUL_, Plasty_AO_, Plasty_PUL_, Plasty, and the Overall group.

### Patient and procedural characteristics

**Figure 4:**
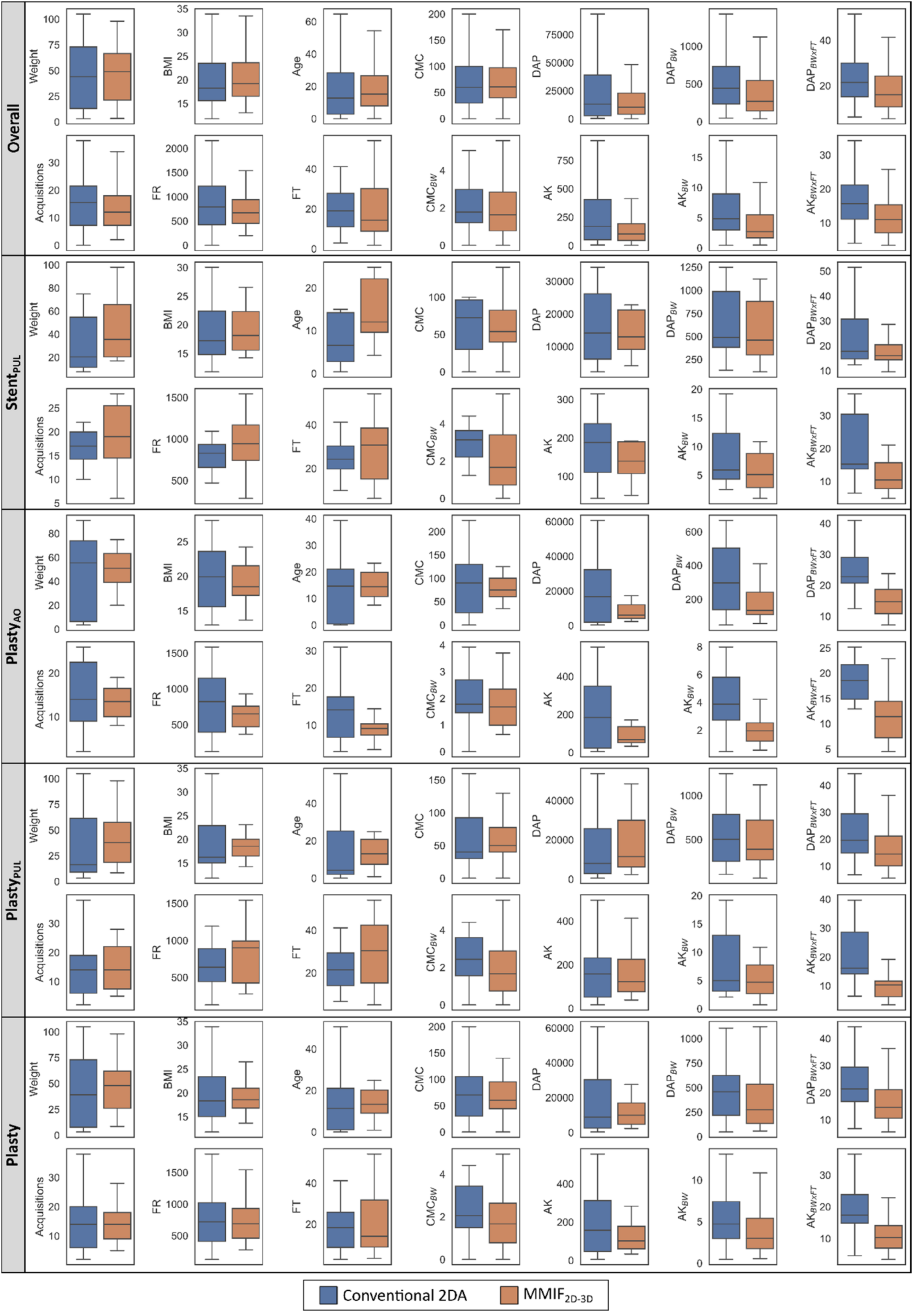
Box plots without outliers of the data in Tables 1, 2.

Patient characteristics are summarized using median and interquartile range (IQR) in Table 1 for the test (MMIF_2D−3D_) and control (Conventional 2DA) cohorts. Table 2 presents cumulative radiation exposure and CMC parameters, together with their corresponding parameters normalized by BW and BW × FT, for all the five (sub)groups. A boxplot representation excluding outliers of Tables 1, 2 is shown in Figure 4. Finally, detailed results of parameter grouping based on irradiation event type and acquisition plane for the Overall group are listed in Table 3. Supplementary Tables S1–S4 provide a similar overview of the parameters grouped by irradiation event and acquisition plane for the matched subgroups.

**Table 1:** Patient characteristics for the Overall group and for four matched subgroups: stenting procedures of the pulmonary artery (Stent_PUL_), angioplasty procedures (balloon dilatation and/or stenting) of the aorta (Plasty_AO_), angioplasty procedures of the pulmonary artery (Plasty_PUL_), and angioplasty procedures of the aorta and/or pulmonary artery (Plasty).

Variable (unit)	Conventional 2DA	MMIF_2D−3D_	*p*-value
Group	Overall (n_2DA_ = 58; n_MMIF_ = 54)		
Weight (kg)	44.0 (13.0–73.0)	49.0 (21.25–**66.5**)	0.637
BMI (kg m^−2^)	18.262 (15.609–23.504)	19.181 (16.525–23.616)	0.438
Age (years)	12.737 (2.817–28.353)	15.259 (7.938–**26.555**)	0.217
Group	Stent_PUL_ (n_2DA_ = 10; n_MMIF_ = 10)		
Weight (kg)	20.5 (11.4–54.875)	35.5 (20.5–65.75)	0.212
BMI (kg m^−2^)	17.257 (14.836–22.393)	18.115 (15.572–**22.315**)	0.597
Age (years)	6.61 (2.817–14.205)	12.027 (9.634–22.125)	0.174
Group	Plasty_PUL_ (n_2DA_ = 19; n_MMIF_ = 19)		
Weight (kg)	16.4 (9.2–61.5)	38.0 (18.6–**57.5**)	0.199
BMI (kg m^−2^)	16.225 (15.05–22.923)	18.553 (16.481–**20.051**)	0.466
Age (years)	4.189 (2.032–25.189)	13.093 (7.419–**20.745**)	0.148
Group	Plasty_AO_ (n_2DA_ = 16; n_MMIF_ = 12)		
Weight (kg)	55.5 (6.5–74.0)	**51.0** (39.125–**63.25**)	0.889
BMI (kg m^−2^)	19.927 (15.591–23.587)	**18.501** (17.238–**21.491**)	0.889
Age (years)	14.636 (0.434–20.99)	**14.485** (10.771–**19.851**)	0.610
Group	Plasty (n_2DA_ = 35; n_MMIF_ = 31)		
Weight (kg)	39.0 (7.85–73.0)	48.0 (26.0–**62.0**)	0.362
BMI (kg m^−2^)	18.321 (15.05–23.388)	18.553 (16.787–**21.006**)	0.684
Age (years)	11.345 (1.108–21.06)	13.293 (9.081–**20.215**)	0.131

n_2DA_, the number of procedures in the matched control group for conventional 2DA; n_MMIF_, the number of procedures in the test group MMIF_2D−3D_.

Bold text indicates a lower median, Q1, or Q3 value in the MMIF_2D−3D_ group compared with the corresponding value encountered in the conventional 2DA group.

**Table 2:** Procedural characteristics, i.e., cumulative radiation exposure parameters and contrast media usage, for the Overall group and for four matched subgroups: stenting procedures of the pulmonary artery (Stent_PUL_), angioplasty procedures (balloon dilatation and/or stenting) of the aorta (Plasty_AO_), angioplasty procedures of the pulmonary artery (Plasty_PUL_), and angioplasty procedures of the aorta and/or pulmonary artery (Plasty).

Group	Variable (Unit)	Conventional 2DA	MMIF_2D−3D_	*p*-value
Overall (n_2DA_ = 58; n_MMIF_ = 54)	Acquisitions (#)	15.5 (7.25–21.5)	**12.0** (7.25–**18.0**)	0.622
	FR (#)	792.0 (423.5–1,221.75)	**671.0** (454.5–**942.0**)	0.507
	FT (min)	19.033 (11.054–27.779)	**14.285** (**8.876**–30.09)	0.169
	DAP (mGy cm^2^)	13,050.336 (2,838.729–39,100.954)	**10,307.832** (4,163.399–**22,888.763**)	0.731
	AK (mGy)	169.68 (52.329–405.202)	**102.297** (**44.624**–**192.634**)	0.188
	CMC (ml)	60.0 (30.0–100.0)	61.0 (40.0–**97.5**)	0.916
	DAP_BW_ (mGy cm^2^ kg^−1^)	442.398 (233.96–733.217)	**268.577** (**143.544**–**545.399**)	0.052
	AK_BW_ (mGy kg^−1^)	4.839 (3.012–8.969)	**2.713** (**1.691**–**5.516**)	0.003
	CMC_BW_ (ml kg^−1^)	1.777 (1.214–2.985)	**1.633** (**0.769**–**2.843**)	0.193
	DAP_BW × FT_ (mGy cm^2^ kg^−1^ min^−1^)	21.459 (15.14–30.037)	**17.818** (**14.113**–**26.849**)	0.264
	AK_BW × FT_ (mGy kg^−1^ min^−1^)	15.709 (11.121–21.183)	**11.653** (**8.965**–**18.474**)	0.018
Stent_PUL_ (n_2DA_ = 10; n_MMIF_ = 10)	Acquisitions (#)	17.0 (14.25–20.0)	19.0 (14.5–25.5)	0.677
	FR (#)	828.5 (658.25–933.5)	945.0 (742.5–1,169.5)	0.496
	FT (min)	24.283 (19.775–30.292)	30.746 (**15.233**–38.54)	0.762
	DAP (mGy cm^2^)	14,167.111 (6,157.926–26,176.615)	**13,017.231** (9,135.732–**21,216.559**)	0.650
	AK (mGy)	187.84 (110.477–237.212)	**139.687** (**107.26**–**190.142**)	0.705
	CMC (ml)	72.5 (30.0–96.25)	**54.0** (40.0–**82.5**)	0.791
	DAP_BW_ (mGy cm^2^ kg^−1^)	487.921 (381.192–985.04)	**459.986** (**300.059**–**880.857**)	0.705
	AK_BW_ (mGy kg^−1^)	5.908 (4.331–12.288)	**5.09** (**2.853**–**8.802**)	0.364
	CMC_BW_ (ml kg^−1^)	3.137 (2.213–3.629)	**1.669** (**0.724**–**3.403**)	0.307
	DAP_BW × FT_ (mGy cm^2^ kg^−1^ min^−1^)	17.795 (14.794–30.741)	18.881 (15.12–**21.172**)	0.762
	AK_BW × FT_ (mGy kg^−1^ min^−1^)	15.303 (13.793–30.407)	**10.759** (**9.169**–**16.726**)	0.131
Plasty_PUL_ (n_2DA_ = 19; n_MMIF_ = 19)	Acquisitions (#)	14.0 (6.0–19.0)	14.0 (7.5–22.0)	0.438
	FR (#)	638.0 (445.0–890.0)	900.0 (**424.5**–994.0)	0.293
	FT (min)	21.5 (14.133–29.417)	30.482 (15.322–42.506)	0.493
	DAP (mGy cm^2^)	8,053.284 (2,905.003–25,738.685)	11,485.319 (6,257.413–29,964.438)	0.389
	AK (mGy)	157.483 (51.774–231.524)	**122.701** (76.205–**225.079**)	0.872
	CMC (ml)	40.0 (30.0–92.5)	50.0 (40.0–**77.5**)	0.693
	DAP_BW_ (mGy cm^2^ kg^−1^)	502.994 (253.158–785.399)	**390.228** (268.577–**721.024**)	0.737
	AK_BW_ (mGy kg^−1^)	4.959 (3.101–12.973)	**4.693** (**2.652**–**7.744**)	0.249
	CMC_BW_ (ml kg^−1^)	2.439 (1.562–3.6)	**1.667** (**0.735**–**2.875**)	0.204
	DAP_BW × FT_ (mGy cm^2^ kg^−1^ min^−1^)	19.605 (14.93–29.486)	**15.232** (**12.058**–**22.598**)	0.249
	AK_BW × FT_ (mGy kg^−1^ min^−1^)	16.111 (14.152–28.6)	**10.481** (**8.019**–**13.761**)	0.006
Plasty_AO_ (n_2DA_ = 16; n_MMIF_ = 12)	Acquisitions (#)	14.0 (9.0–22.5)	**13.5** (10.0–**16.5**)	0.727
	FR (#)	823.0 (395.75–1,149.75)	**652.5** (471.5–**758.0**)	0.318
	FT (min)	14.133 (6.729–17.608)	**9.077** (7.343–**10.355**)	0.150
	DAP (mGy cm^2^)	16,657.327 (1,961.285–32,247.914)	**6,045.466** (4,136.604–**12,019.472**)	0.458
	AK (mGy)	184.257 (23.737–349.611)	**67.496** (52.739–**136.279**)	0.458
	CMC (ml)	90.0 (26.25–130.0)	**75.0** (60.5–**100.0**)	0.816
	DAP_BW_ (mGy cm^2^ kg^−1^)	296.874 (138.95–503.661)	**133.762** (**109.28**–**241.567**)	0.046
	AK_BW_ (mGy kg^−1^)	3.867 (2.72–5.809)	**1.962** (**1.211**–**2.531**)	0.014
	CMC_BW_ (ml kg^−1^)	1.777 (1.463–2.698)	**1.675** (**0.989**–**2.345**)	0.330
	DAP_BW × FT_ (mGy cm^2^ kg^−1^ min^−1^)	22.811 (20.741–29.015)	**16.272** (**14.595**–**25.369**)	0.070
	AK_BW × FT_ (mGy kg^−1^ min^−1^)	18.586 (14.865–21.707)	**12.896** (**9.859**–**16.649**)	0.051
Plasty (n_2DA_ = 35; n_MMIF_ = 31)	Acquisitions (#)	14.0 (6.0–20.0)	14.0 (9.0–**18.0**)	0.718
	FR (#)	719.0 (411.5–1,022.0)	**688.0** (461.0–**933.0**)	0.949
	FT (min)	18.333 (8.708–25.85)	**14.178** (8.884–31.784)	0.974
	DAP (mGy cm^2^)	8,829.225 (2,527.159–30,306.158)	10,007.708 (4,758.84–**16,917.954**)	0.802
	AK (mGy)	157.483 (46.572–313.083)	**102.113** (60.661–**177.917**)	0.500
	CMC (ml)	70.0 (30.0–105.0)	**60.0** (44.0–**95.0**)	0.898
	DAP_BW_ (mGy cm^2^ kg^−1^)	457.349 (218.506–624.543)	**274.384** (**133.762**–**535.505**)	0.225
	AK_BW_ (mGy kg^−1^)	4.72 (2.968–7.42)	**2.988** (**1.77**–**5.421**)	0.037
	CMC_BW_ (ml kg^−1^)	2.062 (1.494–3.438)	**1.667** (**0.783**–**2.636**)	0.114
	DAP_BW × FT_ (mGy cm^2^ kg^−1^ min^−1^)	21.455 (16.82–29.486)	**15.739** (**13.053**–**24.42**)	0.037
	AK_BW × FT_ (mGy kg^−1^ min^−1^)	17.385 (14.862–23.844)	**11.036** (**8.549**–**15.915**)	0.001

n_2DA_, the number of procedures in the matched control group for conventional 2DA; n_MMIF_, the number of procedures in the test group MMIF_2D−3D_; FR, number of acquisition frames; FT, fluoroscopy time; DAP, dose area product; AK, air kerma; CMC, contrast media consumption; DAP_BW_, dose area product normalized by body weight; AK_BW_, air kerma normalized by body weight; CMC_BW_, contrast media consumption normalized by body weight; DAP_BW × FT_, dose area product normalized by the product of body weight and fluoroscopy time; AK_BW × FT_, air kerma normalized by the product of body weight and fluoroscopy time.

Bold text indicates a lower median, Q1, or Q3 value in the MMIF_2D−3D_ group compared with the corresponding value encountered in the conventional 2DA group. Rows with a gray background indicate *p* < 0.05 and therefore statistical significance.

**Table 3:** Radiation exposure parameters grouped by irradiation event type and acquisition plane, listed as median (Q1–Q3) values for the Overall group.

Variable (Unit)	Conventional 2DA (*n* = 58)	MMIF_2D−3D_ (*n* = 54)	*p*-value
DAP^(A)^ (mGy cm^2^)	7,308.795 (1,896.989–25,287.69)	**6,309.346** (2,684.514–**16,094.01**)	0.771
DAP^(B)^ (mGy cm^2^)	3,077.668 (510.902–13,862.318)	**1,625.287** (593.289–**5,090.852**)	0.183
AK^(A)^ (mGy)	73.266 (21.738–201.212)	**54.738** (29.928–**141.209**)	0.736
AK^(B)^ (mGy)	52.559 (12.534–176.807)	**27.553** (**9.285**–**74.665**)	0.069
DAP^F^ (mGy cm^2^)	9,324.848 (2,304.053–26,872.037)	**7,744.786** (2,566.527–**18,033.609**)	0.736
DAP^F(A)^ (mGy cm^2^)	5,477.852 (1,513.517–21,054.668)	**5,464.426** (2,268.587–**14,404.911**)	0.625
DAP^F(B)^ (mGy cm^2^)	1,513.162 (231.323–6,200.844)	**678.634** (**97.773**–**2,192.279**)	0.061
AK^F^ (mGy)	117.267 (45.291–249.087)	**76.248** (**29.58**–**164.302**)	0.130
AK^F(A)^ (mGy)	58.701 (18.129–153.391)	**49.306** (27.514–**127.251**)	0.991
AK^F(B)^ (mGy)	28.591 (3.666–83.415)	**9.945** (**1.709**–**29.81**)	0.022
DAP^A^ (mGy cm^2^)	2,356.695 (358.637–13,457.281)	**2,056.087** (932.948–**5,333.384**)	0.958
DAP^A(A)^ (mGy cm^2^)	949.163 (188.26–4,314.737)	**936.341** (408.824–**2,260.488**)	0.893
DAP^A(B)^ (mGy cm^2^)	641.767 (131.893–6,498.645)	933.456 (294.006–**3,158.306**)	0.935
AK^A^ (mGy)	23.802 (6.591–129.816)	23.839 (11.112–**56.454**)	0.816
AK^A(A)^ (mGy)	8.655 (2.606–26.268)	**7.281** (3.815–**15.565**)	0.650
AK^A(B)^ (mGy)	11.05 (3.177–89.176)	13.559 (4.548–**47.648**)	0.981
FT^(A)^	903.5 (446.25–1,351.5)	**758.266** (**426.171**–1,586.164)	0.958
FT^(B)^	159.5 (42.5–484.75)	**48.461** (**14.531**–**182.944**)	0.006
FR^(A)^	419.5 (220.0–637.0)	**367.5** (242.5–**503.0**)	0.614
FR^(B)^	388.0 (187.25–596.5)	**323.0** (206.0–**464.25**)	0.505
DAP^(A)^_BW_ (mGy cm^2^ kg^−1^)	228.953 (111.164–478.59)	**194.924** (**89.853**–**463.274**)	0.522
DAP^(B)^_BW_ (mGy cm^2^ kg^−1^)	124.627 (40.956–237.84)	**55.681** (**23.987**–**103.098**)	0.005
AK^(A)^_BW_ (mGy kg^−1^)	2.422 (1.314–4.025)	**1.754** (**0.884**–4.415)	0.164
AK^(B)^_BW_ (mGy kg^−1^)	1.988 (0.878–3.454)	**0.841** (**0.375**–**1.7**)	0.001
DAP^F^_BW_ (mGy cm^2^ kg^−1^)	287.961 (161.619–546.347)	**205.032** (**96.407**–**476.367**)	0.077
DAP^F(A)^_BW_ (mGy cm^2^ kg^−1^)	209.729 (79.088–407.343)	**168.418** (**74.944**–436.627)	0.709
DAP^F(B)^_BW_ (mGy cm^2^ kg^−1^)	54.302 (13.682–185.212)	**16.363** (**3.55**–**46.592**)	0.004
AK^F^_BW_ (mGy kg^−1^)	3.462 (1.915–5.88)	**1.868** (**1.082**–**4.843**)	0.007
AK^F(A)^_BW_ (mGy kg^−1^)	1.712 (0.918–3.555)	**1.555** (**0.683**–4.225)	0.319
AK^F(B)^_BW_ (mGy kg^−1^)	0.924 (0.218–2.907)	**0.262** (**0.073**–**0.718**)	0.002
DAP^A^_BW_ (mGy cm^2^ kg^−1^)	62.965 (30.324–192.92)	**46.051** (**27.631**–**109.453**)	0.249
DAP^A(A)^_BW_ (mGy cm^2^ kg^−1^)	30.142 (13.969–64.53)	**20.396** (14.343–**40.217**)	0.256
DAP^A(B)^_BW_ (mGy cm^2^ kg^−1^)	26.207 (12.994–114.462)	**24.265** (13.009–**62.499**)	0.740
AK^A^_BW_ (mGy kg^−1^)	1.157 (0.487–2.072)	**0.619** (**0.355**–**1.313**)	0.048
AK^A(A)^_BW_ (mGy kg^−1^)	0.305 (0.172–0.533)	**0.206** (**0.141**–**0.331**)	0.036
AK^A(B)^_BW_ (mGy kg^−1^)	0.542 (0.263–1.33)	**0.408** (**0.213**–**0.985**)	0.289
DAP^(A)^_BW × FT_ (mGy cm^2^ kg^−1^ min^−1^)	17.569 (12.843–23.272)	**14.661** (**11.101**–**19.876**)	0.145
DAP^(B)^_BW × FT_ (mGy cm^2^ kg^−1^ min^−1^)	36.096 (23.8–70.802)	59.847 (**21.43**–156.891)	0.203
AK^(A)^_BW × FT_ (mGy kg^−1^ min^−1^)	0.163 (0.125–0.234)	**0.138** (**0.103**–**0.191**)	0.046
AK^(B)^_BW × FT_ (mGy kg^−1^ min^−1^)	0.717 (0.391–1.193)	0.947 (**0.367**–3.043)	0.501
DAP^F^_BW × FT_ (mGy cm^2^ kg^−1^ min^−1^)	15.622 (10.479–21.753)	**13.085** (10.499–**20.162**)	0.244
DAP^F(A)^_BW × FT_ (mGy cm^2^ kg^−1^ min^−1^)	13.523 (9.806–19.966)	**11.863** (**9.303**–**17.781**)	0.481
DAP^F(B)^_BW × FT_ (mGy cm^2^ kg^−1^ min^−1^)	21.141 (14.476–28.528)	**17.937** (**11.736**–31.348)	0.688
AK^F^_BW × FT_ (mGy kg^−1^ min^−1^)	0.198 (0.125–0.246)	**0.137** (**0.103**–**0.19**)	0.008
AK^F(A)^_BW × FT_ (mGy kg^−1^ min^−1^)	0.124 (0.095–0.198)	0.125 (**0.08**–**0.17**)	0.259
AK^F(B)^_BW × FT_ (mGy kg^−1^ min^−1^)	0.328 (0.252–0.477)	**0.278** (**0.189**–0.478)	0.176
DAP^A^_BW × FR_ (mGy cm^2^ kg^−1^ fr^−1^)	0.098 (0.064–0.164)	**0.075** (**0.055**–**0.126**)	0.129
DAP^A(A)^_BW × FR_ (mGy cm^2^ kg^−1^ fr^−1^)	0.076 (0.053–0.128)	**0.06** (**0.044**–**0.088**)	0.053
DAP^A(B)^_BW × FR_ (mGy cm^2^ kg^−1^ fr^−1^)	0.11 (0.064–0.167)	**0.099** (**0.057**–0.187)	0.629
AK^A^_BW × FR_ (×10^−3^ mGy kg^−1^ fr^−1^)	1.313 (1.072–2.151)	**1.076** (**0.693**–**1.557**)	0.006
AK^A(A)^_BW × FR_ (×10^−3^ mGy kg^−1^ fr^−1^)	0.843 (0.488–1.162)	**0.569** (**0.349**–**0.828**)	0.008
AK^A(B)^_BW × FR_ (×10^−3^ mGy kg^−1^ fr^−1^)	1.811 (1.404–2.821)	**1.521** (**0.988**–**2.789**)	0.144

DAP, dose area product; AK, air kerma; CMC, contrast media consumption; DAP_BW_, dose area product normalized by body weight; AK_BW_, air kerma normalized by body weight; CMC_BW_, contrast media consumption normalized by body weight; DAP_BW × FT_, dose area product normalized by the product of body weight and fluoroscopy time; AK_BW × FT_, air kerma normalized by the product of body weight and fluoroscopy time.

Bold text indicates a lower median, Q1, or Q3 value in the MMIF_2D−3D_ group compared with the corresponding value encountered in the conventional 2DA group. Rows with a gray background indicate *p* < 0.05 and therefore statistical significance. The first superscript, i.e., either F or A, indicates grouping by fluoroscopy or acquisition, respectively. A missing first superscript refers to the overall (F + A) DAP and AK. The second superscript between brackets, i.e., either (A) or (B), indicates grouping by the frontal or lateral plane, respectively. A missing second superscript refers to the overall [(A) + (B)] DAP and AK of both acquisition planes combined. A missing subscript *** refers to the overall DAP and AK without normalization.

#### Overall group

In terms of patient characteristics, there were no significant differences observed among the Overall group. FT^(B)^ was reduced by 69.6%, resulting in a 71.7% and 69.9% decrease of AK^F(B)^_BW_ and DAP^F(B)^_BW_ and consequently a reduction of AK^(B)^_BW_ (57.7%), DAP^(B)^_BW_ (55.3%), AK^F(B)^ (65.2%), and AK^F^_BW_ (46.1%). Furthermore, the MMIF_2D−3D_ group demonstrated lower values for AK^A(A)^_BW_ (32.4%) and AK^A^_BW_ (46.6%), contributing to an overall decrease in AK_BW_ (43.9%). DAP_BW_ decreased by 39.3%. However, this difference was not statistically significant (*p* = 0.052). AK^F^_BW × FT_ and AK^A(A)^_BW × FR_ exhibited significantly lower values, resulting in a decreased cumulative AK_BW × FT_.

#### Procedure-type subgroups

Significant differences in patient characteristics were not observed among any of the subgroups. In terms of radiation exposure quantities, similar findings to that of the Overall group were noted for the Plasty subgroup, with a 36.7% reduction in AK_BW_. Similarly, the Plasty_AO_ subgroup showed reductions of 49.3% and 54.9% in AK_BW_ and DAP_BW_, respectively. Both the Plasty_AO_ and Plasty subgroups exhibited decreases in AK^F^_BW × FT_ and AK^A(A)^_BW × FR_, while AK^A(B)^_BW × FR_ and eventually AK^A^_BW × FR_ were only lower for the Plasty subgroup. These results led to a notable decrease in both AK_BW × FT_ and DAP_BW × FT_ within both the Plasty_AO_ and Plasty subgroups.

In the remaining two subgroups, Stent_PUL_ and Plasty_PUL_, only some BW_ _× FT or BW × FR normalized quantities showed significantly lower values. For Plasty_PUL_, there were eight exposure quantities showing significant reductions. These included AK^F(A)^_BW × FT_, AK^F(B)^_BW × FT_, and AK^F^_BW × FT_ as well as their associated acquisition counterparts AK^A(A)^_BW × FR_, AK^A(B)^_BW × FR_, and AK^A^_BW × FR_. In addition, AK^(A)^_BW × FT_ and AK_BW × FT_ also exhibited significantly lower values in this subgroup. For Stent_PUL_, only AK^F(B)^_BW × FT_ showed significantly lower values in the test cohort. Although patient characteristics were not significantly different, it is worth noting that patients were, respectively, 1.73 and 1.82 times heavier and 2.32 and 3.13 times older in the Stent_PUL_ and Plasty_PUL_ subgroups.

### Questionnaire

#### Question 1—spared acquisitions

Of the 54 cases, at least one spared acquisition compared with the conventional workflow was reported in 17 cases (31.5% of cases). In these cases, a median of 2.0 (IQR, 2.0–3.0) spared acquisitions were reported, which were considered to be mostly biplane. The cardiologists estimated a corresponding median saved CMC of 18.3 (IQR, 10.0–21.7) ml and a median CMC normalized to body weight of 0.82 (IQR, 0.54–1.40) ml kg^−1^.

#### Question 2—redundant acquisitions

Cardiologists reported a median of 2.0 (IQR, 2.0–3.0) redundant acquisitions in 27 procedures (50.0% of cases). The associated median redundant CMC was estimated at 30.0 (IQR, 23.5–35.8) ml, while the median redundant normalized CMC was 0.63 (IQR, 0.40–0.98) ml kg^−1^.

#### Question 3—relevance of 3D overlay

The cardiologists reported the image fusion to be very useful, with a median score of 4.0 (IQR, 3.0–4.0). None of the cases received a score of 1, indicating that the image fusion was never considered to be misleading.

For 38 cases (70.4%), either spared or redundant acquisitions were reported. There was good agreement among observers regarding the number of spared acquisitions (*κ* = 0.847), and substantial agreement for redundant acquisitions (*κ* = 0.631).

## Discussion

Interventional cardiac catheterization has improved the care of patients with CHD (5). Complex congenital cardiac lesions often require long and even multiple procedures during the lifetime follow-up of patients (9, 29, 30). Therefore, these procedures should be optimized to achieve satisfactory diagnostic and therapeutic yields at as low as reasonably achievable radiation and contrast dose levels (1). The current study assessed the potentially beneficial impact of multimodality 3D image fusion, on radiation and contrast dose.

To the best of our knowledge, this study presents the largest population ever examined to compare radiation and contrast dose in MMIF_2D−3D_-guided procedures vs. conventional biplane 2DA among CHD patients. Furthermore, this is the first study evaluating the effect of MMIF_2D−3D_ on radiation exposure levels grouped by irradiation event type and acquisition plane, exploiting the full potential of the data available inside the DICOM RDSR.

To reach accurate conclusions, we looked at weight-normalized exposure parameters because of the wide range of ages and body weights in the population. The reductions discussed in the following were statistically significant, unless otherwise stated.

Our findings showed a reduced FT^(B)^ in the MMIF_2D−3D_ cohort of the Overall group. This resulted in decreased lateral fluoroscopy-related exposure quantities [AK^F(B)^_BW_ and DAP^F(B)^_BW_], which consequently reduced overall lateral plane exposure [AK^(B)^_BW_ and DAP^(B)^_BW_], and eventually led to a cumulative reduction of AK_BW_. Similarly, AK_BW_ was reduced for the Plasty subgroup, whereas both AK_BW_ and DAP_BW_ decreased in the Plasty_AO_ subgroup. FT^(B)^ can hence be considered the main factor driving the reduction in weight-normalized exposure parameters. In conventional biplane 2DA, a reference angiography is typically performed and used as a roadmap in both planes. This often requires biplane fluoroscopy or switching between frontal and lateral fluoroscopy to navigate guidewires, catheters, and devices to the desired location. However, with the MMIF_2D−3D_ software, a transparent 3D roadmap registered with the frontal plane (unfortunately it cannot register with the lateral plane) is now available. This allows for movement of the frontal C-arm to different projection angles without losing registration of the 3D roadmap. In contrast to conventional biplane 2DA, most of the procedures can be done using the frontal plane without the need of lateral fluoroscopy.

The use of MMIF_2D−3D_ resulted in a reduced FT^(B)^, leading to a decrease in AK_BW_ for the fusion population in the Overall, Plasty_AO_, and Plasty (sub)groups. However, only in the Plasty_AO_ subgroup a DAP_BW_ reduction was shown. This could be due to differences in imaging detector sizes between the frontal and lateral planes. As a result, AK^(B)^ contributes more significantly to cumulative AK compared with DAP^(B)^ contributing to cumulative DAP. Therefore, even though there may be an overall reduction in cumulative AK_BW_ by decreased AK^(B)^_BW_ in the lateral plane [which also leads to decreased DAP^(B)^_BW_], this does not necessarily reduce cumulative DAP_BW_.

Furthermore, the Overall and Plasty (sub)groups show lower AK^F^_BW × FT_ and AK^A^_BW × FR_. The reductions in exposure rate could be the result of several actions taken during the procedure. First, using lower dose imaging protocols in the MMIF_2D−3D_ population may result in lower AK and DAP rates. The availability of MMIF_2D−3D_ allows for lower yet adequate image quality with reduced radiation dosage. Second, utilizing larger field of views (FOVs) instead of the zooming function can lead to lower AK rates, but this also increases x-ray field areas. Because DAP is the product of these parameters and their effects cancel each other out to some extent, the overall impact on average DAP rate will be limited in this case. If operators wish to visualize the full scope of the 3D roadmap, they need to utilize the largest FOV available. Zooming in to a smaller FOV might no longer be necessary. Lastly, the use of less steep angulations in the fusion cohort, such as reducing the use of the lateral plane or employing less steep angulations in the frontal plane, can effectively decrease both AK rate and DAP rate. It is worth noting that the 3D roadmap is always accessible without x-ray exposure. This allows operators to identify the optimal viewing angle even before starting fluoroscopy. Once this optimal angle is determined, operators have the option to choose a less radiation-intensive projection while still ensuring good visibility during subsequent x-ray exposure. In addition, as mentioned earlier, operators mainly shift their focus from the steeper lateral plane to the frontal plane. In the current study, only two DAP quantities normalized by BW × FT or BW × FR were reduced in the Plasty subgroup [DAP^A(A)^_BW × FR_ and DAP_BW × FT_], whereas many BW × FT and BW × FR normalized AK quantities exhibited a decrease, both cumulative and grouped, in multiple (sub)groups. This suggests that the most likely reason for the reduced AK^F^_BW × FT_ and AK^A^_BW × FR_ mentioned previously is the use of larger FOVs.

This study found a reduction in the number of acquisitions and acquisition frames and their associated CMC_BW_, but these differences were not statistically significant. The questionnaire responses also supported these findings. Of the 54 cases, 17 reported at least one spared acquisition, while 27 had redundant acquisitions. This indicates the potential to further reduce radiation exposure and associated CMC. Furthermore, the cardiologists also confirmed the clinical benefit of MMIF_2D−3D_ by scoring its relevance as very useful, with a median value of 4 on a 5-point Likert scale.

The literature presents similar findings regarding cumulative radiation exposure. Table 4 summarizes the key results from relevant studies for comparison purposes. In general, studies comparing CT and MRI image fusion with conventional biplane approaches tend to report lower average or median radiation exposure and contrast volume. Although more than half of the studies in Table 4 reported significantly lower values of one or several radiation exposure and CMC quantities, it is important to note that they primarily focus on a single procedure type and include small patient cohorts (15, 24, 27, 28).

**Table 4:** Summary of the main findings in the literature relevant to the current study.

Author	Populations	Procedure type	Radiation exposure and CMC
Goreczny et al. (27)	MMIF_2D−3D_ (*n* = 7) vs. biplane 2DA (*n* = 8)	PPVR	Significantly lower CMC_BW_ (−60.5%), DAP_BW_ (−66.6%) and AK_BW_ (−68.3%)
Zablah et al. (30)	MMIF_2D−3D_ + optimized radiation settings + DrySeal sheath + ICE^a^ (*n* = 38) vs. biplane 2DA (*n* = 38)	PPVR	Significantly lower FT (−17.1%), CMC_BW_ (−50.0%), DAP_BW_ (−25.0%), and AK (−48.8%)
Goreczny et al. (28)	MMIF_2D−3D_ from MRI (*n* = 14) vs. MMIF_2D−3D_ from CT (*n* = 8)	RVOT Balloon Sizing, PPVR	Significantly lower DAP (−35.0%) and DAP_BW_ (−64.5%) for MRI-guided procedures vs. CT-guided procedures^a^
Goreczny et al. (31)	MMIF_2D−3D_ (*n* = 6) vs. biplane 2DA (*n* = 6)	Pulmonary Vein Interventions	Lower FT (−27%), CMC (−31.5%), DAP (−88.5%), and AK (−82%). The differences were not significant.
Stangenberg et al. (25)	MMIF_2D−3D_ (*n* = 16) vs. biplane 2DA (*n* = 16). BMI matched pairs.	EVAR	Significantly lower FT (−31.3%), CMC (−51.6%), and AK (−39.6%)
Tacher et al. (32)	MMIF_2D−3D_ (*n* = 8) vs. biplane 2DA (*n* = 14)	EVAR	Significantly lower CMC (−72.3%)
Glöckler et al. (15)	3D-guided (MMIF_2D−3D_ or 3DRA) (*n* = 12) vs. biplane 2DA (*n* = 20)	CoA	Significantly lower FT (−18.1%) and lower DAP (−26.4%). DAP not significantly lower.
Arar (33)	MMIF_2D−3D_ (*n* = 7) vs. biplane 2DA (*n* = 17)	CoA	Lower DAP (43.1%). DAP not significantly lower.
Ehret et al. (24)	MMIF_2D−3D_ (*n* = 13) vs. biplane 2DA (*n* = 20)	Aortic Arch Angioplasty	Significantly lower CMC_BW_ (−48.1%), DAP (−51.7%), and FT (−55.9%) (*n*_MMIF _= 13, n_2DA _= 20)
Abu Hazeem et al. (34)	MMIF_2D−3D_ from MRI (*n* = 44) vs. biplane 2DA (*n* = 44). Weight and diagnosis matched pairs	Diagnostic	Significantly lower FT (−14.6%), CMC_BW_ (−39.4%), DAP^F^ (−12.9%), DAP (−37.1%), and AK (−38.6%)

PPVR, percutaneous pulmonary valve replacement; RVOT, right ventricular outflow tract; EVAR, endovascular aortic aneurysm repair; CoA, coarctation aorta.

^a^ Most of the MRI-guided procedures were acknowledged to be performed on a low-dose angiography unit known to significantly reduce radiation exposure during cardiac catheterization in an adult population.

Several publications in the literature compare radiation exposure associated with 3DRA imaging vs. conventional biplane 2DA (35). Some studies show higher radiation exposure with 3DRA, yet more recent research suggests lower values when using optimized exposure settings for the 3DRA imaging protocol. Some authors argue that diagnostic 3DRA is outdated for primary diagnosis and 3D guidance due to the availability of MMIF_2D−3D_ (24, 36). However, it remains valuable when preprocedural imaging is not possible (36).

## Limitations

This study has several limitations. First, there are a limited number of available MMIF_2D−3D_ cases. The procedures involved diverse procedure types and complexities, including patients with and without preprocedural surgery and catheterizations, as well as a wide range of patient characteristics owing to the long-term follow-up. This poses challenges in achieving meaningful matching between the test and control groups. For example, Stent_PUL_ and Plasty_PUL_ subpopulations show higher patient weight and age, yet these differences were not statistically significant. Moreover, Stent_PUL_ was the smallest procedure-type subgroup with different number of lesions, whether or not pretreated. Even though procedures were regrouped by similar procedure types, variations in patient-specific factors and technical challenges can significantly influence the imaging protocol and the projection views chosen by the operators. These choices will determine how the dose rate varies by patient thickness, fluoroscopy time, number of frames used, collimation, and FOV, and hence the eventual cumulative radiation exposure of the procedure. Nevertheless, referenced literature deals with similar limitations, while often reporting smaller sample sizes than the current study.

Furthermore, the reasons for the lower weight-normalized AK and DAP rates could not be confirmed as information regarding the use of lower dose imaging protocols and larger FOVs was unavailable in the DICOM RDSR.

Regarding the questionnaires, the numbers of the spared and redundant acquisitions were in line with the lower median number of frames and acquisitions in the MMIF_2D−3D_ group. However, this evaluation is subjective and there was no statistically significant difference in the number of frames and acquisitions between the MMIF_2D−3D_ and conventional 2DA cohorts.

To enable multimodality image fusion, a preprocedural cardiac CT or MRI is required. In this study, 30 cases were CT-guided and 24 were MRI-guided. The radiation exposure from the preprocedural CT should be considered for cumulative radiation exposure. However, organ or effective doses (OD or ED) are needed for both modalities to calculate the total radiation dose delivered to the patient. In this study, organ and effective doses were not assessed as the study did not impact clinical routine: MMIF_2D−3D_ was only used when preprocedural CT or MRI was available without requesting additional imaging when unavailable. For the cardiac catheterizations included in this study, organ and effective doses could potentially be calculated using previously established DAP_BW_-to-ED conversion factors, which were determined for the same x-ray imaging system as the current study (13). Finally, the current study was performed in a single center and was not randomized.

## Conclusion

MMIF_2D−3D_ enables significant radiation exposure reduction in a single-center matched CHD population. It moves the focus of the operator toward the frontal plane, obviating the need for most of the lateral fluoroscopy exposure, hence reducing FT^(B)^ and ultimately AK_BW_. Subgroup analysis similarly showed significant AK_BW_ reduction in the Plasty and Plasty_AO_ subgroups. DAP_BW_ was significantly lower in the Plasty_AO_ subgroup. No significantly lower CMC_BW_ was observed in the MMIF_2D−3D_ population for any of the analyzed (sub)groups.

## Data Availability

An anonymous dataset containing the cumulative exposure information from the RDSRs of all included procedures and supporting the conclusions of this article, can be made available by the authors upon reasonable request.
