# Supplementary material for: Multimodality 3D image fusion with live fluoroscopy reduces radiation dose during catheterization of congenital heart defects
Source: Front Cardiovasc Med. 2024 Jan 11;10:1292039. doi: 10.3389/fcvm.2023.1292039 (PMC10808650; doi:10.3389/fcvm.2023.1292039)
Supplement: Supplementary file 1 [file Datasheet1.pdf]

Table S1

Variable	Conventional 2DA (n=10)	MMIF$_{2D\text{-}3D}$ (n=10)	P-value
DAP$^{(A)}$ [mGy.cm^2]	13084.874 (4608.992 - 22713.57)	**11958.494** (7374.414 - **18958.854**)	0.821
DAP$^{(B)}$ [mGy.cm^2]	1254.948 (436.646 - 2941.734)	1774.063 (1030.78 - **2582.988**)	0.326
AK$^{(A)}$ [mGy]	124.237 (83.924 - 204.904)	128.869 (95.788 - **166.212**)	0.940
AK$^{(B)}$ [mGy]	26.069 (11.155 - 48.842)	**23.912** (15.136 - **34.446**)	0.880
DAP^{F} [mGy.cm^2]	12346.777 (5534.58 - 22423.571)	**11191.202** (7271.743 - **19035.196**)	0.762
DAP$^{F(A)}$ [mGy.cm^2]	12011.089 (4259.439 - 20843.651)	**10776.999** (6741.043 - **17829.129**)	0.880
DAP$^{F(B)}$ [mGy.cm^2]	335.688 (118.548 - 1158.127)	930.992 (192.334 - 1466.404)	0.450
AK^{F} [mGy]	135.072 (89.266 - 192.132)	**130.981** (**85.479** - **166.104**)	0.880
AK$^{F(A)}$ [mGy]	111.561 (76.441 - 185.097)	124.363 (84.954 - **152.253**)	1.000
AK$^{F(B)}$ [mGy]	7.02 (2.779 - 15.792)	12.47 (**2.575** - 19.421)	0.597
DAP^{A} [mGy.cm^2]	1855.674 (517.302 - 3477.763)	2195.587 (1504.964 - **2731.922**)	0.705
DAP$^{A(A)}$ [mGy.cm^2]	837.021 (279.191 - 2097.043)	1042.066 (556.988 - **1815.544**)	0.705
DAP$^{A(B)}$ [mGy.cm^2]	341.632 (216.382 - 1122.7)	1023.629 (725.223 - 1246.174)	0.151
AK^{A} [mGy]	25.826 (12.027 - 45.08)	**22.404** (19.525 - **30.221**)	0.880
AK$^{A(A)}$ [mGy]	7.746 (5.665 - 23.473)	8.644 (6.546 - **15.736**)	0.821
AK$^{A(B)}$ [mGy]	7.33 (6.15 - 20.949)	14.743 (10.071 - **16.345**)	0.290
FT$^{(A)}$	1409.5 (1135.0 - 1690.25)	1763.443 (**898.782** - 2179.238)	0.597
FT$^{(B)}$	63.5 (30.25 - 122.0)	69.358 (41.38 - 148.174)	0.762
FR$^{(A)}$	422.5 (337.5 - 536.75)	481.5 (371.25 - 584.75)	0.880
FR$^{(B)}$	356.0 (304.0 - 444.25)	463.5 (371.25 - 584.75)	0.226
DAP$^{(A)}_{BW}$ [mGy.cm^2.kg^{-1}]	418.356 (337.111 - 853.384)	**416.455** (**279.525** - **814.981**)	0.545
DAP$^{(B)}_{BW}$ [mGy.cm^2.kg^{-1}]	66.534 (28.912 - 88.377)	**59.845** (34.115 - 88.724)	0.940
AK$^{(A)}_{BW}$ [mGy.kg^{-1}]	4.663 (3.629 - 10.738)	4.73 (**2.392** - **7.08**)	0.450
AK$^{(B)}_{BW}$ [mGy.kg^{-1}]	1.065 (0.644 - 1.937)	**0.831** (**0.431** - **1.148**)	0.406
DAP$^{F}_{BW}$ [mGy.cm^2.kg^{-1}]	417.079 (299.414 - 901.127)	421.89 (**243.733** - **757.713**)	0.597
DAP$^{F(A)}_{BW}$ [mGy.cm^2.kg^{-1}]	387.31 (295.116 - 778.547)	400.424 (**242.247** - **717.461**)	0.705
DAP$^{F(B)}_{BW}$ [mGy.cm^2.kg^{-1}]	19.138 (6.111 - 44.947)	26.285 (8.069 - **43.651**)	0.880
AK$^{F}_{BW}$ [mGy.kg^{-1}]	4.664 (3.511 - 10.806)	**4.473** (**2.285** - **7.297**)	0.496
AK$^{F(A)}_{BW}$ [mGy.kg^{-1}]	4.132 (3.375 - 9.499)	4.423 (**2.135** - **6.726**)	0.496
AK$^{F(B)}_{BW}$ [mGy.kg^{-1}]	0.297 (0.103 - 1.236)	0.345 (0.111 - **0.572**)	0.545
DAP$^{A}_{BW}$ [mGy.cm^2.kg^{-1}]	64.093 (53.605 - 101.988)	**61.232** (**45.742** - 131.482)	0.821
DAP$^{A(A)}_{BW}$ [mGy.cm^2.kg^{-1}]	33.648 (24.406 - 57.662)	**30.145** (**20.41** - **51.582**)	0.705
DAP$^{A(B)}_{BW}$ [mGy.cm^2.kg^{-1}]	23.399 (21.479 - 28.354)	30.884 (**17.854** - 55.213)	0.545
AK$^{A}_{BW}$ [mGy.kg^{-1}]	1.227 (0.88 - 1.428)	**0.753** (**0.539** - 1.511)	0.257
AK$^{A(A)}_{BW}$ [mGy.kg^{-1}]	0.472 (0.314 - 0.707)	**0.308** (**0.194** - **0.493**)	0.199
AK$^{A(B)}_{BW}$ [mGy.kg^{-1}]	0.608 (0.375 - 0.779)	**0.427** (**0.287** - **0.712**)	0.597
DAP$^{(A)}_{BWxFT}$ [mGy.cm^2.kg^{-1}.min^{-1}]	16.453 (13.264 - 30.659)	17.127 (**11.718** - **19.048**)	0.650
DAP$^{(B)}_{BWxFT}$ [mGy.cm^2.kg^{-1}.min^{-1}]	34.097 (28.725 - 70.802)	46.177 (**24.426** - 106.556)	0.870
AK$^{(A)}_{BWxFT}$ [mGy.kg^{-1}.min^{-1}]	0.218 (0.203 - 0.446)	**0.139** (**0.109** - **0.256**)	0.151
AK$^{(B)}_{BWxFT}$ [mGy.kg^{-1}.min^{-1}]	0.946 (0.783 - 1.193)	**0.737** (**0.33** - 1.413)	0.514
DAP$^{F}_{BWxFT}$ [mGy.cm^2.kg^{-1}.min^{-1}]	14.955 (11.277 - 29.239)	**14.845** (**11.194** - **17.69**)	0.406
DAP$^{F(A)}_{BWxFT}$ [mGy.cm^2.kg^{-1}.min^{-1}]	14.973 (11.272 - 29.691)	14.97 (**11.077** - **17.329**)	0.597
DAP$^{F(B)}_{BWxFT}$ [mGy.cm^2.kg^{-1}.min^{-1}]	17.402 (13.119 - 23.552)	**15.228** (**12.294** - **18.541**)	0.568
AK$^{F}_{BWxFT}$ [mGy.kg^{-1}.min^{-1}]	0.199 (0.182 - 0.451)	**0.131** (**0.108** - **0.233**)	0.174
AK$^{F(A)}_{BWxFT}$ [mGy.kg^{-1}.min^{-1}]	0.189 (0.18 - 0.385)	**0.127** (**0.094** - **0.226**)	0.151
AK$^{F(B)}_{BWxFT}$ [mGy.kg^{-1}.min^{-1}]	0.287 (0.263 - 0.529)	**0.198** (**0.167** - **0.227**)	0.034
DAP$^{A}_{BWxFR}$ [mGy.cm^2.kg^{-1}.fr^{-1}]	0.077 (0.063 - 0.091)	**0.076** (**0.057** - 0.115)	0.940
DAP$^{A(A)}_{BWxFR}$ [mGy.cm^2.kg^{-1}.fr^{-1}]	0.071 (0.062 - 0.08)	0.077 (0.066 - 0.102)	0.880
DAP$^{A(B)}_{BWxFR}$ [mGy.cm^2.kg^{-1}.fr^{-1}]	0.074 (0.052 - 0.11)	0.08 (0.055 - **0.104**)	0.568
AK$^{A}_{BWxFR}$ [x 10^{-3} mGy.kg^{-1}.fr^{-1}]	1.217 (0.999 - 2.066)	**0.882** (**0.706** - **1.318**)	0.070
AK$^{A(A)}_{BWxFR}$ [x 10^{-3} mGy.kg^{-1}.fr^{-1}]	1.010 (0.938 - 1.703)	**0.726** (**0.531** - **1.105**)	0.290
AK$^{A(B)}_{BWxFR}$ [x 10^{-3} mGy.kg^{-1}.fr^{-1}]	1.495 (1.373 - 1.863)	**1.086** (**0.778** - **1.263**)	0.072

Table S2

Variable	Conventional 2DA (n=16)	MMIF$_{2D-3D}$ (n=12)	P-value
DAP$^{(A)}$ [mGy.cm^2]	5789.748 (834.252 - 12758.035)	**4300.208** (2401.658 - **6724.367**)	0.926
DAP$^{(B)}$ [mGy.cm^2]	7234.234 (305.986 - 17690.909)	**1974.694** (1577.732 - **3323.522**)	0.307
AK$^{(A)}$ [mGy]	56.552 (13.264 - 119.539)	**40.082** (24.334 - **67.166**)	0.781
AK$^{(B)}$ [mGy]	90.236 (5.211 - 214.491)	**29.09** (24.956 - **56.632**)	0.286
DAP^{F} [mGy.cm^2]	9101.004 (1739.098 - 20830.363)	**4230.644** (2526.925 - **8179.2**)	0.516
DAP$^{F(A)}$ [mGy.cm^2]	3749.727 (776.107 - 7888.111)	**3641.142** (1844.341 - **5610.667**)	0.816
DAP$^{F(B)}$ [mGy.cm^2]	2623.497 (185.709 - 5909.124)	**607.12** (303.062 - **1485.145**)	0.114
AK^{F} [mGy]	96.616 (21.228 - 186.973)	**45.734** (24.563 - **90.214**)	0.246
AK$^{F(A)}$ [mGy]	36.778 (10.95 - 74.744)	**33.68** (20.515 - **55.749**)	0.781
AK$^{F(B)}$ [mGy]	34.014 (3.388 - 71.428)	**8.389** (4.036 - **20.003**)	0.086
DAP^{A} [mGy.cm^2]	5180.491 (237.969 - 14586.176)	**2327.005** (1192.534 - **3004.364**)	0.781
DAP$^{A(A)}$ [mGy.cm^2]	1753.07 (117.065 - 3400.646)	**688.762** (502.992 - **1052.293**)	0.353
DAP$^{A(B)}$ [mGy.cm^2]	1932.339 (126.717 - 8402.153)	**1634.581** (610.545 - **2209.166**)	0.889
AK^{A} [mGy]	53.766 (3.935 - 170.731)	**26.518** (19.223 - **44.635**)	0.816
AK$^{A(A)}$ [mGy]	17.581 (1.643 - 33.271)	**6.767** (4.746 - **9.279**)	0.330
AK$^{A(B)}$ [mGy]	23.19 (2.035 - 106.7)	**22.378** (11.312 - **34.577**)	0.781
FT$^{(A)}$	491.5 (283.0 - 682.25)	**431.603** (375.548 - **583.054**)	0.577
FT$^{(B)}$	105.0 (56.75 - 298.0)	**30.729** (**13.014** - **71.909**)	0.011
FR$^{(A)}$	411.5 (230.0 - 650.25)	**326.5** (235.75 - **385.75**)	0.236
FR$^{(B)}$	388.0 (187.75 - 513.5)	**323.0** (235.75 - **372.25**)	0.416
DAP$^{(A)}_{BW}$ [mGy.cm^2.kg^{-1}]	130.109 (85.955 - 199.805)	**91.266** (**79.067** - **138.469**)	0.348
DAP$^{(B)}_{BW}$ [mGy.cm^2.kg^{-1}]	161.366 (38.72 - 254.708)	**42.796** (**27.324** - **95.269**)	0.084
AK$^{(A)}_{BW}$ [mGy.kg^{-1}]	1.53 (1.074 - 2.3)	**1.173** (**0.739** - **1.384**)	0.167
AK$^{(B)}_{BW}$ [mGy.kg^{-1}]	1.987 (1.16 - 3.06)	**0.578** (**0.447** - **1.355**)	0.043
DAP$^{F}_{BW}$ [mGy.cm^2.kg^{-1}]	209.186 (99.66 - 297.547)	**99.503** (**74.35** - **153.357**)	0.026
DAP$^{F(A)}_{BW}$ [mGy.cm^2.kg^{-1}]	91.51 (67.527 - 115.446)	**75.67** (**63.98** - 117.97)	0.693
DAP$^{F(B)}_{BW}$ [mGy.cm^2.kg^{-1}]	37.154 (22.398 - 169.417)	**10.621** (**6.029** - **28.992**)	0.026
AK$^{F}_{BW}$ [mGy.kg^{-1}]	2.322 (1.24 - 3.38)	**1.168** (**0.75** - **1.669**)	0.034
AK$^{F(A)}_{BW}$ [mGy.kg^{-1}]	1.025 (0.69 - 1.515)	**0.99** (**0.615** - **1.102**)	0.587
AK$^{F(B)}_{BW}$ [mGy.kg^{-1}]	0.873 (0.348 - 1.97)	**0.145** (**0.095** - **0.412**)	0.014
DAP$^{A}_{BW}$ [mGy.cm^2.kg^{-1}]	97.195 (26.424 - 185.095)	**42.787** (33.51 - **67.252**)	0.324
DAP$^{A(A)}_{BW}$ [mGy.cm^2.kg^{-1}]	28.512 (13.234 - 53.805)	**17.063** (**11.99** - **20.26**)	0.139
DAP$^{A(B)}_{BW}$ [mGy.cm^2.kg^{-1}]	35.272 (12.756 - 133.895)	**24.198** (22.47 - **46.848**)	0.767
AK$^{A}_{BW}$ [mGy.kg^{-1}]	1.321 (0.544 - 2.203)	**0.568** (**0.41** - **0.911**)	0.139
AK$^{A(A)}_{BW}$ [mGy.kg^{-1}]	0.295 (0.197 - 0.542)	**0.158** (**0.108** - **0.229**)	0.023
AK$^{A(B)}_{BW}$ [mGy.kg^{-1}]	0.666 (0.333 - 1.702)	**0.431** (**0.32** - **0.7**)	0.521
DAP$^{(A)}_{BWxFT}$ [mGy.cm^2.kg^{-1}.min^{-1}]	17.066 (13.995 - 19.747)	**13.812** (**12.45** - **17.136**)	0.139
DAP$^{(B)}_{BWxFT}$ [mGy.cm^2.kg^{-1}.min^{-1}]	42.118 (26.531 - 82.976)	108.352 (28.879 - 228.718)	0.324
AK$^{(A)}_{BWxFT}$ [mGy.kg^{-1}.min^{-1}]	0.173 (0.15 - 0.201)	**0.141** (**0.111** - **0.161**)	0.076
AK$^{(B)}_{BWxFT}$ [mGy.kg^{-1}.min^{-1}]	0.697 (0.422 - 1.543)	1.484 (**0.417** - 3.585)	0.278
DAP$^{F}_{BWxFT}$ [mGy.cm^2.kg^{-1}.min^{-1}]	15.795 (10.447 - 18.775)	**11.297** (10.765 - **15.5**)	0.256
DAP$^{F(A)}_{BWxFT}$ [mGy.cm^2.kg^{-1}.min^{-1}]	11.447 (9.282 - 13.652)	**11.002** (10.052 - 14.574)	0.921
DAP$^{F(B)}_{BWxFT}$ [mGy.cm^2.kg^{-1}.min^{-1}]	22.823 (19.326 - 31.656)	**18.274** (**12.403** - 33.228)	0.554
AK$^{F}_{BWxFT}$ [mGy.kg^{-1}.min^{-1}]	0.2 (0.118 - 0.244)	**0.134** (**0.112** - **0.161**)	0.049
AK$^{F(A)}_{BWxFT}$ [mGy.kg^{-1}.min^{-1}]	0.11 (0.088 - 0.167)	0.119 (0.092 - **0.139**)	0.961
AK$^{F(B)}_{BWxFT}$ [mGy.kg^{-1}.min^{-1}]	0.305 (0.264 - 0.459)	**0.284** (**0.2** - **0.414**)	0.402
DAP$^{A}_{BWxFR}$ [mGy.cm^2.kg^{-1}.fr^{-1}]	0.12 (0.074 - 0.187)	**0.075** (**0.059** - **0.111**)	0.167
DAP$^{A(A)}_{BWxFR}$ [mGy.cm^2.kg^{-1}.fr^{-1}]	0.075 (0.049 - 0.125)	**0.048** (**0.045** - **0.065**)	0.103
DAP$^{A(B)}_{BWxFR}$ [mGy.cm^2.kg^{-1}.fr^{-1}]	0.119 (0.077 - 0.202)	**0.112** (**0.069** - **0.159**)	0.657
AK$^{A}_{BWxFR}$ [x 10^{-3} mGy.kg^{-1}.fr^{-1}]	1.639 (1.117 - 2.214)	**1.076 (0.905 - 1.226)**	0.152
AK$^{A(A)}_{BWxFR}$ [x 10^{-3} mGy.kg^{-1}.fr^{-1}]	0.968 (0.500 - 1.372)	**0.485 (0.418 - 0.664)**	0.026
AK$^{A(B)}_{BWxFR}$ [x 10^{-3} mGy.kg^{-1}.fr^{-1}]	2.012 (1.413 - 3.864)	**1.592 (1.232 - 2.072)**	0.554

Table S3

**Variable**	**Conventional 2DA (n=19)**	**MMIF$_{2D-3D}$ (n=19)**	**P-value**
DAP$^{(A)}$ [mGy.cm^2]	6052.48 (2225.957 - 22146.004)	10197.373 (4912.389 - 24377.414)	0.274
DAP$^{(B)}$ [mGy.cm^2]	1225.845 (447.954 - 3592.681)	**1158.589** (806.0 - 4084.656)	0.474
AK$^{(A)}$ [mGy]	78.283 (37.229 - 168.624)	104.757 (47.252 - **158.019**)	0.630
AK$^{(B)}$ [mGy]	27.571 (10.752 - 64.14)	29.879 (12.111 - **52.483**)	0.895
DAP^{F} [mGy.cm^2]	7711.476 (2434.312 - 22356.463)	10136.37 (5404.599 - 25672.858)	0.439
DAP$^{F(A)}$ [mGy.cm^2]	5453.637 (2086.901 - 20540.992)	9939.97 (4531.21 - 22664.202)	0.314
DAP$^{F(B)}$ [mGy.cm^2]	463.247 (221.376 - 1714.873)	795.356 (**193.689** - 3167.245)	0.781
AK^{F} [mGy]	125.803 (45.123 - 199.756)	**100.756** (61.592 - **173.134**)	0.782
AK$^{F(A)}$ [mGy]	70.393 (32.226 - 160.161)	89.83 (43.194 - **148.086**)	0.609
AK$^{F(B)}$ [mGy]	12.3 (3.687 - 45.795)	14.476 (**2.64** - **40.628**)	0.759
DAP^{A} [mGy.cm^2]	618.808 (375.466 - 3418.128)	2063.05 (985.938 - **3047.26**)	0.439
DAP$^{A(A)}$ [mGy.cm^2]	317.393 (212.647 - 2059.261)	983.403 (477.457 - 2170.424)	0.457
DAP$^{A(B)}$ [mGy.cm^2]	220.934 (102.169 - 930.237)	899.3 (301.976 - 1265.335)	0.111
AK^{A} [mGy]	13.142 (6.912 - 42.085)	22.225 (12.268 - **29.615**)	0.550
AK$^{A(A)}$ [mGy]	6.263 (2.762 - 16.041)	7.483 (4.835 - **15.466**)	0.569
AK$^{A(B)}$ [mGy]	6.137 (2.686 - 14.472)	12.397 (5.113 - 17.052)	0.214
FT$^{(A)}$	1118.0 (681.0 - 1575.5)	1477.364 (909.227 - 2133.625)	0.148
FT$^{(B)}$	128.0 (51.5 - 335.5)	**91.462** (**42.895** - **173.857**)	0.373
FR$^{(A)}$	393.0 (239.0 - 504.0)	450.0 (296.0 - 506.0)	0.381
FR$^{(B)}$	299.0 (107.0 - 443.5)	450.0 (170.0 - 496.5)	0.157
DAP$^{(A)}_{BW}$ [mGy.cm^2.kg^{-1}]	365.987 (154.326 - 522.644)	**364.192** (201.217 - 643.604)	0.872
DAP$^{(B)}_{BW}$ [mGy.cm^2.kg^{-1}]	79.422 (31.914 - 177.172)	**61.025** (**26.08** - **109.006**)	0.405
AK$^{(A)}_{BW}$ [mGy.kg^{-1}]	3.778 (1.736 - 8.826)	4.362 (**1.729** - **5.537**)	0.569
AK$^{(B)}_{BW}$ [mGy.kg^{-1}]	1.638 (0.732 - 2.753)	**0.833** (**0.386** - **1.799**)	0.118
DAP$^{F}_{BW}$ [mGy.cm^2.kg^{-1}]	439.7 (214.21 - 676.968)	**362.013** (227.492 - **613.221**)	0.672
DAP$^{F(A)}_{BW}$ [mGy.cm^2.kg^{-1}]	302.411 (140.401 - 490.563)	354.999 (173.674 - 584.158)	0.849
DAP$^{F(B)}_{BW}$ [mGy.cm^2.kg^{-1}]	49.635 (9.161 - 152.34)	**36.068** (**6.966** - **53.385**)	0.300
AK$^{F}_{BW}$ [mGy.kg^{-1}]	3.816 (2.558 - 12.136)	4.366 (**2.192** - **6.42**)	0.300
AK$^{F(A)}_{BW}$ [mGy.kg^{-1}]	3.423 (1.519 - 7.718)	4.098 (**1.5** - **5.204**)	0.672
AK$^{F(B)}_{BW}$ [mGy.kg^{-1}]	1.195 (0.229 - 2.488)	**0.504** (**0.123** - **0.704**)	0.118
DAP$^{A}_{BW}$ [mGy.cm^2.kg^{-1}]	54.558 (28.706 - 70.842)	**47.106** (**28.684** - 113.77)	0.942
DAP$^{A(A)}_{BW}$ [mGy.cm^2.kg^{-1}]	31.117 (16.996 - 37.362)	**22.87** (**15.445** - 50.993)	0.988
DAP$^{A(B)}_{BW}$ [mGy.cm^2.kg^{-1}]	22.721 (8.257 - 25.813)	**20.514** (13.796 - 53.508)	0.373
AK$^{A}_{BW}$ [mGy.kg^{-1}]	0.837 (0.481 - 1.4)	**0.582** (**0.335** - **1.255**)	0.389
AK$^{A(A)}_{BW}$ [mGy.kg^{-1}]	0.303 (0.17 - 0.598)	**0.282** (**0.143** - **0.397**)	0.373
AK$^{A(B)}_{BW}$ [mGy.kg^{-1}]	0.361 (0.178 - 0.635)	**0.326** (0.202 - 0.694)	0.942
DAP$^{(A)}_{BWxFT}$ [mGy.cm^2.kg^{-1}.min^{-1}]	17.575 (13.364 - 29.406)	**14.774** (**10.766** - **19.657**)	0.157
DAP$^{(B)}_{BWxFT}$ [mGy.cm^2.kg^{-1}.min^{-1}]	28.215 (19.178 - 34.097)	31.944 (20.326 - 66.617)	0.668
AK$^{(A)}_{BWxFT}$ [mGy.kg^{-1}.min^{-1}]	0.228 (0.189 - 0.371)	**0.132** (**0.085** - **0.211**)	0.019
AK$^{(B)}_{BWxFT}$ [mGy.kg^{-1}.min^{-1}]	0.758 (0.373 - 0.946)	**0.429** (**0.306** - 0.982)	0.373
DAP$^{F}_{BWxFT}$ [mGy.cm^2.kg^{-1}.min^{-1}]	15.653 (11.328 - 26.639)	**13.482** (**9.662** - **18.539**)	0.166
DAP$^{F(A)}_{BWxFT}$ [mGy.cm^2.kg^{-1}.min^{-1}]	15.708 (11.336 - 26.843)	**13.022** (**9.576** - **17.463**)	0.226
DAP$^{F(B)}_{BWxFT}$ [mGy.cm^2.kg^{-1}.min^{-1}]	17.402 (12.448 - 23.552)	**14.307** (**8.198** - **21.713**)	0.373
AK$^{F}_{BWxFT}$ [mGy.kg^{-1}.min^{-1}]	0.218 (0.184 - 0.41)	**0.135** (**0.096** - **0.2**)	0.005
AK$^{F(A)}_{BWxFT}$ [mGy.kg^{-1}.min^{-1}]	0.195 (0.168 - 0.327)	**0.122** (**0.068** - **0.199**)	0.024
AK$^{F(B)}_{BWxFT}$ [mGy.kg^{-1}.min^{-1}]	0.329 (0.263 - 0.529)	**0.195** (**0.171** - **0.294**)	0.009
DAP$^{A}_{BWxFR}$ [mGy.cm^2.kg^{-1}.fr^{-1}]	0.086 (0.063 - 0.126)	**0.059** (**0.053** - **0.108**)	0.274
DAP$^{A(A)}_{BWxFR}$ [mGy.cm^2.kg^{-1}.fr^{-1}]	0.078 (0.059 - 0.143)	**0.071** (**0.047** - **0.099**)	0.184
DAP$^{A(B)}_{BWxFR}$ [mGy.cm^2.kg^{-1}.fr^{-1}]	0.074 (0.052 - 0.11)	0.079 (**0.05** - **0.107**)	0.959
AK$^{A}_{BWxFR}$ [x 10^{-3} mGy.kg^{-1}.fr^{-1}]	1.310 (1.069 - 2.127)	**0.867** (**0.694** - **1.143**)	0.005
AK$^{A(A)}_{BWxFR}$ [x 10^{-3} mGy.kg^{-1}.fr^{-1}]	1.059 (0.939 - 1.661)	**0.577** (**0.374** - **1.029**)	0.019
AK$^{A(B)}_{BWxFR}$ [x 10^{-3} mGy.kg^{-1}.fr^{-1}]	1.551 (1.373 - 2.010)	**1.113** (**0.764** - **1.359**)	0.034

Table S4

Variable	Conventional 2DA (n=35)	$MMIF_{2D-3D}$ (n=31)	Pval
$DAP^{(A)}$ [$mGy.cm^2$]	6052.48 (2025.324 - 19511.715)	6185.265 (2917.254 - **14439.45**)	0.386
$DAP^{(B)}$ [$mGy.cm^2$]	1766.706 (377.571 - 8677.362)	1877.115 (953.974 - **4082.327**)	0.913
$AK^{(A)}$ [mGy]	72.927 (25.246 - 153.721)	**56.27** (38.467 - **128.869**)	0.872
$AK^{(B)}$ [mGy]	46.52 (9.488 - 130.992)	**29.794** (15.367 - **54.378**)	0.468
DAP^{F} [$mGy.cm^2$]	8117.318 (2116.787 - 22182.442)	**7753.477** (3058.759 - **13594.051**)	0.802
$DAP^{F(A)}$ [$mGy.cm^2$]	4565.123 (1606.507 - 13967.481)	5084.536 (2625.468 - **12706.191**)	0.278
$DAP^{F(B)}$ [$mGy.cm^2$]	1389.754 (179.024 - 3499.066)	**795.356** (193.689 - **2048.894**)	0.365
AK^{F} [mGy]	114.408 (35.294 - 199.756)	**83.347** (41.967 - **130.981**)	0.393
$AK^{F(A)}$ [mGy]	48.473 (21.744 - 111.561)	49.564 (30.733 - 120.371)	0.525
$AK^{F(B)}$ [mGy]	19.24 (3.542 - 58.045)	**10.465** (**2.81** - **28.488**)	0.152
DAP^{A} [$mGy.cm^2$]	1815.58 (306.422 - 10051.51)	2063.05 (1122.727 - **3094.282**)	0.621
$DAP^{A(A)}$ [$mGy.cm^2$]	791.742 (157.655 - 2583.77)	**711.936** (477.457 - **1438.943**)	0.812
$DAP^{A(B)}$ [$mGy.cm^2$]	320.882 (103.058 - 2791.753)	1079.647 (501.506 - **1955.43**)	0.167
AK^{A} [mGy]	21.022 (5.758 - 111.084)	25.094 (13.522 - **36.524**)	0.621
$AK^{A(A)}$ [mGy]	7.89 (2.538 - 23.235)	**7.08** (4.49 - **12.099**)	0.903
$AK^{A(B)}$ [mGy]	6.189 (2.233 - 33.622)	14.764 (7.478 - **28.416**)	0.201
$FT^{(A)}$	746.0 (395.0 - 1152.0)	794.241 (430.974 - 1731.189)	0.332
$FT^{(B)}$	118.0 (51.5 - 335.5)	**55.998** (**23.364** - **168.153**)	0.044
$FR^{(A)}$	393.0 (225.0 - 572.0)	**346.0** (258.5 - **466.5**)	0.847
$FR^{(B)}$	319.0 (165.5 - 461.5)	344.0 (217.0 - **460.0**)	0.572
$DAP^{(A)}_{BW}$ [$mGy.cm^2.kg^{-1}$]	193.48 (111.56 - 413.797)	198.122 (**87.451** - 457.829)	0.903
$DAP^{(B)}_{BW}$ [$mGy.cm^2.kg^{-1}$]	94.296 (35.461 - 229.731)	**54.471** (**27.018** - **98.899**)	0.040
$AK^{(A)}_{BW}$ [$mGy.kg^{-1}$]	1.928 (1.303 - 4.564)	**1.8** (**0.888** - 4.742)	0.400
$AK^{(B)}_{BW}$ [$mGy.kg^{-1}$]	1.642 (0.905 - 3.043)	**0.828** (**0.401** - **1.475**)	0.007
DAP^{F}_{BW} [$mGy.cm^2.kg^{-1}$]	277.99 (135.955 - 493.481)	**221.017** (**97.66** - 497.53)	0.295
$DAP^{F(A)}_{BW}$ [$mGy.cm^2.kg^{-1}$]	130.055 (79.422 - 379.908)	162.804 (**72.765** - 439.803)	0.893
$DAP^{F(B)}_{BW}$ [$mGy.cm^2.kg^{-1}$]	40.13 (13.94 - 174.059)	**26.558** (**6.713** - **43.471**)	0.030
AK^{F}_{BW} [$mGy.kg^{-1}$]	3.188 (1.87 - 5.795)	**1.808** (**1.099** - **4.755**)	0.071
$AK^{F(A)}_{BW}$ [$mGy.kg^{-1}$]	1.569 (0.879 - 3.616)	**1.556** (**0.758** - 4.423)	0.648
$AK^{F(B)}_{BW}$ [$mGy.kg^{-1}$]	0.909 (0.229 - 2.198)	**0.319** (**0.101** - **0.589**)	0.009
DAP^{A}_{BW} [$mGy.cm^2.kg^{-1}$]	57.875 (27.127 - 143.113)	**44.586** (28.684 - **86.941**)	0.407
$DAP^{A(A)}_{BW}$ [$mGy.cm^2.kg^{-1}$]	29.168 (14.2 - 43.055)	**19.278** (**13.689** - **35.563**)	0.266
$DAP^{A(B)}_{BW}$ [$mGy.cm^2.kg^{-1}$]	23.073 (10.695 - 43.425)	23.536 (15.187 - 53.508)	0.630
AK^{A}_{BW} [$mGy.kg^{-1}$]	1.007 (0.527 - 1.585)	**0.582** (**0.345** - **1.106**)	0.079
$AK^{A(A)}_{BW}$ [$mGy.kg^{-1}$]	0.303 (0.174 - 0.598)	**0.193** (**0.121** - **0.327**)	0.030
$AK^{A(B)}_{BW}$ [$mGy.kg^{-1}$]	0.418 (0.273 - 0.828)	**0.39** (**0.22** - **0.694**)	0.594
$DAP^{(A)}_{BWxFT}$ [$mGy.cm^2.kg^{-1}.min^{-1}$]	17.562 (13.645 - 22.138)	**13.848** (**11.519** - **18.815**)	0.052
$DAP^{(B)}_{BWxFT}$ [$mGy.cm^2.kg^{-1}.min^{-1}$]	31.267 (23.872 - 65.278)	39.436 (**20.326** - 114.461)	0.457
$AK^{(A)}_{BWxFT}$ [$mGy.kg^{-1}.min^{-1}$]	0.202 (0.151 - 0.306)	**0.136** (**0.098** - **0.179**)	0.002
$AK^{(B)}_{BWxFT}$ [$mGy.kg^{-1}.min^{-1}$]	0.717 (0.391 - 1.193)	**0.524** (**0.323** - 1.617)	0.912
DAP^{F}_{BWxFT} [$mGy.cm^2.kg^{-1}.min^{-1}$]	15.653 (10.952 - 19.832)	**11.862** (**10.584** - **17.43**)	0.071
$DAP^{F(A)}_{BWxFT}$ [$mGy.cm^2.kg^{-1}.min^{-1}$]	12.523 (10.397 - 20.417)	**12.133** (**9.792** - **17.203**)	0.338
$DAP^{F(B)}_{BWxFT}$ [$mGy.cm^2.kg^{-1}.min^{-1}$]	19.655 (14.476 - 25.958)	**16.063** (**11.017** - 26.397)	0.259
AK^{F}_{BWxFT} [$mGy.kg^{-1}.min^{-1}$]	0.205 (0.159 - 0.319)	**0.135** (**0.103** - **0.17**)	0.001
$AK^{F(A)}_{BWxFT}$ [$mGy.kg^{-1}.min^{-1}$]	0.18 (0.099 - 0.236)	**0.122** (**0.071** - **0.153**)	0.037
$AK^{F(B)}_{BWxFT}$ [$mGy.kg^{-1}.min^{-1}$]	0.313 (0.263 - 0.477)	**0.214** (**0.174** - **0.352**)	0.012
DAP^{A}_{BWxFR} [$mGy.cm^2.kg^{-1}.fr^{-1}$]	0.093 (0.064 - 0.156)	**0.067** (**0.053** - **0.114**)	0.082
$DAP^{A(A)}_{BWxFR}$ [$mGy.cm^2.kg^{-1}.fr^{-1}$]	0.076 (0.057 - 0.131)	**0.058** (**0.045** - **0.083**)	0.029
$DAP^{A(B)}_{BWxFR}$ [$mGy.cm^2.kg^{-1}.fr^{-1}$]	0.092 (0.064 - 0.142)	**0.081** (**0.053** - **0.125**)	0.626
AK^{A}_{BWxFR} [x 10^{-3} $mGy.kg^{-1}.fr^{-1}$]	1.444 (1.069 - 2.206)	**0.958** (**0.704** - **1.143**)	0.001
$AK^{A(A)}_{BWxFR}$ [x 10^{-3} $mGy.kg^{-1}.fr^{-1}$]	1.029 (0.562 - 1.582)	**0.574** (**0.372** - **0.745**)	0.001
$AK^{A(B)}_{BWxFR}$ [x 10^{-3} $mGy.kg^{-1}.fr^{-1}$]	1.650 (1.373 - 2.630)	**1.283** (**0.820** - **1.774**)	0.044

Supplementary Table legends

Table S1

Radiation exposure parameters grouped by irradiation event type and acquisition plane, listed as median (Q1-Q3) values for the subgroup $Stent_{PUL}$. Bold text indicates a lower median, Q1 or Q3 value in the $MMIF_{2D-3D}$ group compared to the corresponding value encountered in the conventional 2DA group. Rows with a grey background indicate $p < 0.05$ and therefore statistical significance. Abbreviations: cfr. Table 2 and Table 3.

Table S2

Radiation exposure parameters grouped by irradiation event type and acquisition plane, listed as median (Q1-Q3) values for the subgroup $Plasty_{AO}$. Bold text indicates a lower median, Q1 or Q3 value in the $MMIF_{2D-3D}$ group compared to the corresponding value encountered in the conventional 2DA group. Rows with a grey background indicate $p < 0.05$ and therefore statistical significance. Abbreviations: cfr. Table 2 and Table 3.

Table S3

Radiation exposure parameters grouped by irradiation event type and acquisition plane, listed as median (Q1-Q3) values for the subgroup $Plasty_{PUL}$. Bold text indicates a lower median, Q1 or Q3 value in the $MMIF_{2D-3D}$ group compared to the corresponding value encountered in the conventional 2DA group. Rows with a grey background indicate $p < 0.05$ and therefore statistical significance. Abbreviations: cfr. Table 2 and Table 3.

Table S4

Radiation exposure parameters grouped by irradiation event type and acquisition plane, listed as median (Q1-Q3) values for the subgroup Plasty. Bold text indicates a lower median, Q1 or Q3 value in the $MMIF_{2D-3D}$ group compared to the corresponding value encountered in the conventional 2DA group. Rows with a grey background indicate $p < 0.05$ and therefore statistical significance. Abbreviations: cfr. Table 2 and Table 3.
